# Stigmatizing Language in Gender-Expansive Patient Records: Corpus Development, Disparity Analysis, and Natural Language Processing–Based Detection Study

**DOI:** 10.2196/91089

**Published:** 2026-08-27

**Authors:** Liyang Xue, Mary Chayko, Vivek Kumar Singh

**Affiliations:** 1 Department of Library and Information Science Rutgers, The State University of New Jersey New Brunswick, NJ United States

**Keywords:** AI, algorithmic fairness, artificial intelligence, EHR, electronic health record, health equity, natural language processing, NLP, stigmatizing language

## Abstract

**Background:**

Stigmatizing language (SL) in electronic health records (EHRs) can influence clinical decision-making, propagate bias across care encounters, and undermine patient trust. Gender-expansive patients (GEPs) may be particularly vulnerable to documentation-based stigma; however, large-scale quantitative evidence and fairness-aware evaluation of automated SL detection methods remain limited.

**Objective:**

This study aims to construct a gender-expansive-inclusive EHR corpus, quantify demographic disparities in SL using parallel outcome definitions with and without misgendering, and evaluate fairness-aware natural language processing (NLP) methods for automated detection of SL.

**Methods:**

We developed an annotated corpus of 754 clinical notes from the MIMIC-IV (Medical Information Mart for Intensive Care IV) database, including 366 GEP notes and 388 matched nongender-expansive patient (NGEP) notes, labeled for SL and its subtypes. Parallel outcome definitions were constructed with and without misgendering. Multivariable logistic regression was used to assess associations between gender-expansive status and SL while adjusting for race, age, and primary language. Multiple NLP models were evaluated for SL detection. Fairness-aware post hoc threshold optimization based on equalized odds principles was applied using training-set predictions to reduce subgroup error disparities.

**Results:**

SL was identified in 62.3% (228/366) of GEP notes compared with 25.5% (99/388) of NGEP notes. When misgendering was excluded, the prevalence remained higher among gender-expansive notes at 41.8% (153/366). In multivariable models, gender-expansive status was strongly associated with stigmatizing documentation when misgendering was included (adjusted odds ratio [OR] 4.87, 95% CI 3.54-6.70) and remained significant when misgendering was excluded (adjusted OR 2.12, 95% CI 1.54-2.91). Post hoc equalized odds threshold optimization for a state-of-the-art transformer-based detector reduced the difference in false-positive rate (ΔFPR) from 15.76 to 6.65 percentage points (pp) and the difference in true-positive rate (ΔTPR) from 7.22 pp to substantially lower levels while maintaining similar accuracy (82.78%-83.44%). When misgendering was excluded, fairness optimization reduced ΔFPR to 2.98 pp and ΔTPR to 0.65 pp, with an overall accuracy of 88.08%.

**Conclusions:**

SL is common in EHR documentation and disproportionately affects GEPs, and automated detection models show persistent subgroup performance gaps. Disparities remained significant even when misgendering was excluded, indicating that bias extends beyond identity-specific errors to broader evaluative language. This study introduces the first annotated corpus focused on SL in GEP documentation, quantifies demographic disparities, and demonstrates practical fairness-aware NLP strategies that can reduce error-rate inequities while preserving accuracy. These findings support equity-focused interventions to address SL through fairness-aware models as assistive auditing tools with human oversight.

## Introduction

Gender-expansive patients (GEPs) are individuals whose gender identity or expression exists outside, or expands beyond, the conventional male/female binary, including transgender, nonbinary, gender-queer, agender, and gender-fluid identities [1-3]. Research suggests that they can experience persistent discrimination and invalidation in health systems, leading to delayed care, reduced trust, and poorer health outcomes [4,5]. In clinical settings, these harms may be amplified by the use of stigmatizing language (SL) in electronic health records (EHRs). SL includes negative descriptors, expressions of doubt, misgendering, deadnaming, and other identity-invalidating phrasing that implicitly or explicitly conveys bias, blame, or diminished credibility toward a patient [6-9]. Misgendering and related linguistic invalidation are common among GEPs and are associated with adverse psychological outcomes and reduced engagement with care [10-12]. Because EHR notes are routinely viewed, copied, and reused across encounters, language that misrepresents or delegitimizes a patient’s identity can propagate widely and influence a number of care decisions, screening recommendations, and continuity of care [11,13,14].

Accurate EHR representation of gender identity is therefore critical for equitable care. However, empirical studies reveal inconsistent or incomplete capture of key identity fields and frequent identity-related inaccuracies in EHR notes [10,13-15]. National informatics guidance from the US Office of the National Coordinator for Health Information Technology (ONC) and the World Professional Association for Transgender Health (WPATH) recommends structured collection and display of information related to sex assigned at birth, gender identity, pronouns, and chosen names to support identity-affirming documentation and reduce misgendering [15-19]. Despite these recommendations, implementation remains uneven across health systems and EHR platforms, reflecting persistent structural and workflow barriers [14,15,18,19]. As health systems increasingly rely on digital documentation and data-driven decision tools, improving linguistic respect and identity fidelity in EHR notes is foundational to patient safety and equity.

Although stigma in health care has been documented, research on SL directed toward GEPs remains limited. Existing studies are primarily qualitative or based on very small samples, often including fewer than 100 cases [6-12]. To date, there are no large-scale quantitative analyses, no publicly available annotated datasets, and little evaluation of automated detection methods in this domain. This lack of systematic evidence limits the ability to quantify linguistic harms and develop scalable, equity-focused interventions for GEPs.

Recent advances in natural language processing (NLP) have emerged as a potential pathway for the automated identification and mitigation of SL in clinical documentation. Early work established lexicon-supported pipelines for detecting negative descriptors and credibility- or compliance-related stigma [6,20]. Transformer-based models, including BERT (Bidirectional Encoder Representations from Transformers), ClinicalBERT, and Longformer, can capture more subtle contextual patterns and have revealed systematic disparities in linguistic framing across conditions and demographic groups [6,7,20-24]. These findings underscore the need for subgroup-aware evaluation when deploying NLP tools in real-world documentation workflows, particularly in settings involving historically marginalized patient groups.

However, prior NLP research has not examined whether automated SL detection models themselves exhibit differential error patterns for GEPs. Existing systems rarely incorporate formal fairness criteria or evaluate false-positive and false-negative disparities across protected groups. This omission is consequential, as misclassification may disproportionately burden marginalized patients when models are used to flag documentation or trigger downstream interventions. Moreover, fairness frameworks such as equalized odds and group-specific decision thresholding have not been applied to SL detection in EHRs [25,26]. As a result, fairness-aware SL detection for GEPs remains largely unexplored.

To address these gaps, we constructed a GEP-focused EHR dataset using targeted keyword filtering and manual annotation of 3 established stigma subtypes: credibility and obstinacy, compliance, and descriptors. In addition to these subtypes, we also annotated misgendering, which is a uniquely consequential identity-related harm under descriptors [11,12]. Because misgendering may disproportionately influence descriptor-level stigma in gender-expansive documentation, we conducted parallel analyses with and without misgendering included in the outcome definition to distinguish identity-specific errors from broader evaluative language. We quantified demographic disparities in SL and evaluated multiple NLP-based SL models, including a recent multistage transfer learning (MSTL) framework based on transformer architecture for automated detection [20]. Further, we designed a fairness-aware modeling strategy that treats GEP status as a protected attribute and evaluates subgroup differences in model error rates. Drawing on equalized odds principles, we implemented a post hoc, group-specific thresholding approach to identify accuracy-preserving configurations that reduce false-positive rate (FPR) disparities. To our knowledge, this study provides the first integrated dataset, disparity analysis, and fairness-constrained modeling framework for detecting SL directed toward GEPs in EHRs, establishing a foundation for equitable clinical NLP.

## Methods

### Ethical Considerations

This study used the publicly available MIMIC-IV (Medical Information Mart for Intensive Care IV) database containing deidentified EHR data [27,28]. Access to the dataset requires completion of human subjects research training and execution of a data use agreement through PhysioNet.

This study was reviewed and approved by the Rutgers University Institutional Review Board (study ID Pro2025000546) with an exempt determination for secondary analysis of deidentified data. The study used fully deidentified records from the MIMIC-IV database and involved no direct interaction with human participants. The institutional review board granted a waiver of informed consent because the research involved minimal risk and used existing deidentified data. The original MIMIC-IV data collection was approved by the institutional review boards of Beth Israel Deaconess Medical Center and the Massachusetts Institute of Technology, with a waiver of informed consent [27].

### Dataset Construction and Annotation Process

The dataset was derived from deidentified EHR notes in the MIMIC-IV database [27]. MIMIC-IV is a large, publicly accessible EHR dataset sourced from patients admitted to Beth Israel Deaconess Medical Center in Boston, Massachusetts, with records spanning hospital and emergency department encounters between 2008 and 2019. It includes comprehensive clinical information covering diverse patient demographics, diagnoses, treatments, and deidentified free-text notes, with all protected health information removed and dates shifted to comply with Health Insurance Portability and Accountability Act (HIPAA) Safe Harbor deidentification standards [27,28]. To identify documentation related to GEPs, we applied a targeted keyword-based filtering strategy adapted from prior work showing that transgender and gender-diverse patients can be identified in EHR data using keyword-based approaches when structured gender identity data are incomplete [29-31]. Keywords included transgender, trans man, trans woman, nonbinary, gender-fluid, gender-queer, and related terms referencing affirmed gender identity or pronoun use. The complete keyword list is provided in section S1 of Multimedia Appendix 1.

**Figure 1:**
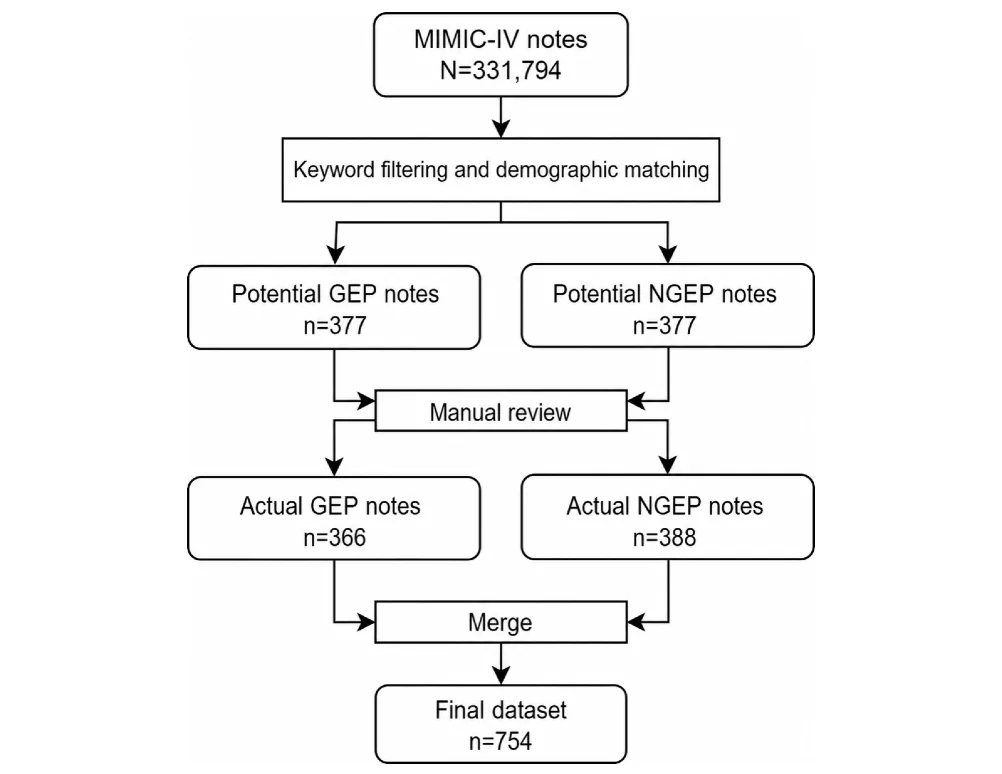
Cohort identification and dataset construction process for GEP and NGEP notes. GEP: gender-expansive patient; MIMIC-IV: Medical Information Mart for Intensive Care IV; NGEP: nongender-expansive patient.

All notes in the MIMIC-IV corpus (N=331,794) were first screened using a keyword-based filtering strategy to identify candidate notes potentially corresponding to GEPs. During the same filtering stage, a comparable set of candidate nongender-expansive patient (NGEP) notes was selected to approximate the marginal distribution of race, age, and primary language observed in the candidate GEP cohort. This process yielded 377 candidate GEP notes and 377 candidate NGEP notes. All candidate notes were then manually reviewed to confirm whether gender-related terms referred to the patient. During this process, notes initially identified as potential GEPs were reclassified as NGEP if the gender-related terms referred to other individuals (eg, family members, templated content, or unrelated contexts), and notes that did not meet the inclusion criteria were excluded. After manual review and reclassification, 366 GEP notes and 388 NGEP notes met the inclusion criteria, resulting in the final analytic dataset of 754 notes (Figure 1). Reclassified NGEP notes containing gender-related keywords were retained in the overall corpus. Retaining these notes preserved clinically realistic lexical contexts and avoided creating an artificially separable comparison group in which gender-related vocabulary appeared only among GEP notes.

The final analytic dataset used in all analyses consisted of 754 notes following iterative refinement of keyword filtering and cohort construction. Because this identification strategy relies on explicit identity-related language appearing in clinical documentation, it may preferentially capture notes in which gender identity is visible or actively discussed. Consequently, the resulting GEP cohort should be interpreted as a keyword-identified documentation cohort rather than a representative sample of all GEPs in the underlying population.

Matching of NGEP notes was designed to improve demographic comparability at the cohort level rather than to enforce exact one-to-one balance within each demographic subgroup. The variables of race, age, and primary language were considered during candidate selection to approximate the distribution observed in the GEP cohort, but exact matching across all strata was not required. Gender was intentionally excluded as a matching variable because structured EHR gender fields for GEPs often reflect sex assigned at birth rather than affirmed gender identity, which can introduce opportunities for misclassification [11,12]. In the NGEP cohort, the structured EHR gender field indicated the presence of both male and female patients. Because this field does not reliably reflect affirmed gender identity for GEPs and may introduce systematic misclassification, gender was not included as a matching variable or covariate in subsequent analyses. As a result, differences in gender composition within the NGEP cohort could not be fully controlled and may represent a source of residual confounding. Addressing this limitation would require datasets with validated gender identity fields and a different cohort construction strategy, which was beyond the scope of the present study and is a suggestion for future research.

Two coders (LX and VKS) conducted multiple alignment rounds using EHR notes outside the final dataset to refine category definitions and develop the annotation codebook (section S2 of Multimedia Appendix 1). Annotators were instructed to evaluate each note comprehensively and assign multiple stigma subtypes where applicable. The process was repeated until overall Cohen κ exceeded 0.80 and percentage agreement surpassed 90%, indicating strong interannotator reliability. During full annotation, notes were independently reviewed, with approximately 10% double-annotated to assess reliability. Reliability was further supported through comparison with a prior gold-standard SL dataset (n=24; Cohen κ=0.78; 91.7% agreement) [6]. Each note was labeled for the presence or absence of 3 stigma subtypes following definitions from prior work: credibility and obstinacy, compliance, and descriptors [6]. Table 1 contains a summary of the 3 stigma subtypes and examples.

**Table 1:** Stigmatizing language subtypes and examples. Examples are drawn from EHRa notes in the MIMIC-IV-Noteb dataset [27]. The subtypes are not mutually exclusive.

Categories	Definitions	Examples
Credibility and obstinacy	Language that challenges the patient’s reliability or depicts the patient as resistant, uncooperative, or difficult	threatening staff, threatening kids; completely out of control.
Compliance	Statements suggesting nonadherence to prescribed treatment or medical recommendations	got treated but left AMA; medication non-compliance.
Descriptors	Statements describing the patient’s behavior, attitude, or manner of acting	knocked over some soda to get their attention; Pt agitated.
Misgendering	Pronoun or gendered reference inconsistent with the patient’s affirmed gender indicated in the same note	transgender woman...she...he...male...her

^a^EHR: electronic health record.

^b^MIMIC-IV-Note: Medical Information Mart for Intensive Care IV, clinical notes module.

Gender identity was also annotated, and all GEP notes were additionally assessed for misgendering, defined as the use of pronouns, names, or gendered descriptors inconsistent with affirmed gender identity [11,12]. Affirmed gender identity was determined based on explicit statements in the clinical note, such as documented gender identity, pronoun specification, or consistent gendered references throughout the note. Misgendering was annotated only when the note contained clear evidence of affirmed gender identity and the clinician used pronouns, names, or gendered descriptors inconsistent with that identity. Medically necessary anatomical or organ-specific documentation was not coded as misgendering unless it was paired with an incongruent gendered label or identity-invalidating phrasing. Notes without sufficient context to determine affirmed gender identity were not labeled as misgendering.

Because misgendering may disproportionately influence descriptor prevalence in gender-expansive documentation, we additionally created a parallel descriptor variable that excluded misgendering. All subsequent analyses were conducted using both descriptor definitions to evaluate whether observed disparities were driven primarily by misgendering or by other evaluative descriptors. Annotation was conducted at the note level, meaning that a note was labeled as stigmatizing if it contained at least 1 instance of any stigma subtype. This design supports document-level auditing and model development for long EHR notes but does not capture within-note variation in the frequency or intensity of stigmatizing expressions.

### Multivariable Analysis of SL Across Demographics and Subtypes

To examine demographic correlates of SL and assess whether GEPs were disproportionately described using stigmatizing terms, a series of multivariable logistic regression analyses were conducted. All models were estimated using both outcome definitions, one including misgendering within descriptor-related stigma and one excluding misgendering, to assess the extent to which disparities depended on identity-specific errors vs broader evaluative language. The dependent variable represented the presence of SL within each note (1=stigmatizing; 0=nonstigmatizing), with GEP status as the primary predictor. Covariates included patient race, primary language, and age group.

Age was modeled as an ordinal variable (0 to 3) based on quartiles of the analytic sample (n=754; median 46, IQR 29-62 years), corresponding to ages 18-29 years, 30-46 years, 47-62 years, and 63-91 years. Race and language were treated as categorical predictors, with White race and English language as reference groups. To address sparse observations in certain race categories and ensure stable estimation, low-frequency race categories were combined into an “other” category for regression analyses. Odds ratios (ORs) were thus interpretable as relative likelihoods of SL compared with these majority categories.

Two models were estimated: an unadjusted model including only GEP status and a fully adjusted model incorporating age, race, and language. Changes in the GEP OR between models were used to assess demographic confounding. To examine effect heterogeneity, additional models tested interactions between GEP status and each demographic factor (language, age, and race). Interaction terms were included to examine potential intersectional disparities, as prior literature in health disparities research suggests that clinical outcomes and documentation patterns may vary across overlapping demographic identities and that interaction models are appropriate for evaluating such joint effects [8,32,33]. Because several subgroup cells were small, particularly among non-English-speaking and Asian GEP notes, these interaction analyses were treated as exploratory and interpreted cautiously. Section S3 of Multimedia Appendix 1 provides additional model details.

Stigma subtypes were also analyzed as separate binary outcomes using identical predictors. Descriptor-related stigma was analyzed using two definitions: (1) descriptors including misgendering and (2) descriptors excluding misgendering. This parallel specification allowed us to evaluate whether disparities in descriptor-related stigma were primarily driven by misgendering or reflected broader patterns of evaluative language directed toward GEPs.

To further assess the robustness of the association between GEP status and SL, we conducted repeated balanced-sampling sensitivity analyses. In this procedure, GEP and NGEP notes were repeatedly sampled in equal proportions while also enforcing matched distributions across all demographic covariates included in the regression models, including race, primary language, and age group. Sampling was performed using stratified balancing so that the marginal distributions of these variables were comparable between groups in each run. Logistic regression models were then estimated across multiple independent runs using the same predictor structure as in the primary analyses. Two parallel sensitivity analyses were conducted: one including misgendering within the descriptor category and one excluding misgendering. This procedure ensured that the observed associations were not driven by imbalances in group size or demographic composition and allowed us to evaluate the stability of the results across balanced samples and alternative definitions of descriptor-level stigma. As an additional sensitivity analysis for the primary regression models, notes with race coded as Asian, other, or unknown were excluded to assess robustness to alternative handling of low-frequency race categories.

All analyses were implemented in Python using the *statsmodels* package. Model convergence and multicollinearity were verified across specifications. Subtype-specific ORs with 95% CIs were reported.

### Benchmarking NLP Models for Detecting SL Across GEPs and NGEPs

To evaluate automated detection of SL and compare performance across GEP and NGEP groups, we implemented a suite of benchmark models spanning classical machine learning classifiers and transformer-based architectures. Seven models were implemented to represent a continuum from traditional classifiers to advanced transformer-based architectures: logistic regression, support vector machine, random forest, multinomial naive Bayes, BERT [22], ClinicalBERT [23], and Longformer [24].

Classical models were trained using term frequency–inverse document frequency or count-based features. Transformer-based models were included to assess the benefit of pretrained contextual representations for EHR notes. Because EHR notes frequently exceed the 512-token input limit of standard transformer architectures, BERT and ClinicalBERT were applied using sliding-window chunking. Longformer models processed sequences of up to 4096 tokens. We also evaluated an MSTL framework previously developed for SL detection [20]. In this framework, the model undergoes sequential adaptation across 3 stages to progressively refine representations of SL in EHR notes: semantic adaptation, syntactic adaptation, and task-specific adaptation. To prevent direct note-level overlap, notes included in the keyword-identified analytic dataset for the present study were excluded from the corpus used during MSTL pretraining. This exclusion was performed at the note level rather than the patient level. Therefore, the exact analytic notes, including held-out test notes, were not used during MSTL pretraining. However, other notes from the same patients may have been present in the broader pretraining corpus.

### Data Splitting and Hyperparameter Optimization

The dataset was partitioned into training and testing sets using an 80/20 split. This train/test split was performed at the note level rather than the patient level. Stratified sampling was used to preserve proportional representation of stigma labels and demographic variables used in subgroup analyses, including GEP status, race, primary language, and age group. All model training, hyperparameter tuning, and threshold optimization procedures were performed exclusively on the training data. Classical machine learning models were optimized using grid search within the training set. Transformer-based models were fine-tuned using the AdamW optimizer with binary cross-entropy loss [34]. The held-out test set was reserved strictly for final model evaluation and was not used during model training, hyperparameter selection, or threshold optimization. All experiments were implemented in Python using scikit-learn for classical models and the Hugging Face Transformers library for neural models. All experiments were executed in Google Colab using an NVIDIA A100 GPU with 80 GB of VRAM to support training of long-sequence transformer models.

For models using sliding-window chunking, long documents were divided into sequential text segments compatible with the model’s maximum input length. Each chunk generated a probability estimate for SL. Chunk-level probabilities were aggregated by taking the maximum predicted probability across all chunks to produce a single document-level probability for each note. For subtype-specific experiments, model configurations were selected using validation performance. Models were trained on the training set and evaluated using standard metrics, including accuracy, precision, recall, *F*_1_-score, and area under the receiver operating characteristic curve (ROC-AUC). To better account for class imbalance, we additionally report the area under the precision-recall curve (PR-AUC), which provides a more informative assessment of performance for the positive class. For each subtype, one configuration was selected to maximize overall accuracy, and an alternative configuration was selected to maximize the *F*_1_-score to account for class imbalance. These optimized configurations were then applied to the held-out test set for final evaluation. Results reported in section S4 of Multimedia Appendix 1 reflect these different optimization objectives rather than separate model architectures.

### Equalized Odds-Constrained Threshold Optimization

To evaluate and mitigate fairness disparities in automated SL detection, we compared 4 transformer-based classifiers under an equalized odds framework. Equalized odds requires comparable true-positive rates (TPRs) and FPRs across protected groups. Rather than enforcing strict equality, we quantified disparities by computing absolute differences in TPR and FPR (ΔTPR and ΔFPR, respectively) between GEP and NGEP groups, with lower values indicating more equitable error distributions.

For each model, prediction probabilities were first generated for the training data. A grid-based threshold search was conducted using prediction probabilities generated on the training set after model fitting. No separate validation set or cross-validation folds were used for threshold selection. Thresholds were selected exclusively based on training-set predictions and then applied without modification to the held-out test set for final evaluation to avoid information leakage. Two threshold configurations were derived: an accuracy-optimized configuration, defined as the threshold pair that maximized overall classification accuracy on the training data, and a fairness-optimized configuration, defined as the threshold pair that minimized ΔFPR between groups while maintaining strong overall predictive performance. Equalized odds-based fairness metrics evaluate disparities in both FPRs and TPRs, but prior work has shown that the choice of which error type to prioritize should depend on the intended application and the potential harms associated with misclassification. Because the present model is designed for auditing and documentation quality monitoring rather than direct clinical decision-making, unequal FPRs may lead to disproportionate flagging of documentation for certain demographic groups. Therefore, ΔFPR was selected as the primary fairness objective while maintaining overall predictive performance [25,35,36]. Once thresholds were selected using the training data, they were applied without modification to the held-out test set to compute the final performance and fairness metrics. This procedure avoids information leakage from the test data and preserves the validity of the evaluation.

## Results

### Dataset Overview and Annotation Reliability

Annotation quality was established through multiple coder alignment rounds and reached good agreement, with an overall Cohen κ of 0.85 and an overall percentage agreement of 92.5%. A total of 754 EHR notes were analyzed, including 327 stigmatized and 427 nonstigmatized notes. The dataset was nearly balanced between GEPs and NGEPs. Three primary subtypes of stigma were annotated at the note level, along with a dedicated flag for misgendering. Subtype labels were not mutually exclusive, meaning that a single note could contain more than 1 stigma subtype. All stigmatized notes contained at least 1 subtype label. Table 2 summarizes the dataset composition and the distribution of these subtypes across patient groups. Within the GEP subset, 74.88% (152/203) of descriptor-labeled notes contained explicit misgendering. Misgendering therefore constituted the dominant form of descriptor-level stigma.

**Table 2:** Dataset composition and stigma subtype distribution.

Metric	Frequency, n/N (%)
Total notes^a^	754/754 (100.00)
Stigmatized notes (with misgendering)	327/754 (43.37)
Stigmatized notes (excluding misgendering)	252/754 (33.42)
GEPs^b^	228/366 (62.30)
NGEPs^c^	99/388 (25.52)
Descriptors: GEP (with misgendering)	203/366 (55.46)
Descriptors: GEP (excluding misgendering subtype)^d^	102/366 (27.87)
Descriptors: NGEP	55/388 (14.18)
Compliance: GEP	102/366 (27.87)
Compliance: NGEP	48/388 (12.37)
Credibility and obstinacy: GEP	85/366 (23.22)
Credibility and obstinacy: NGEP	48/388 (12.37)
Misgendering (GEP only)	152/366 (41.53)

^a^Percentages for all-note and stigmatized-note rows use the 754-note denominator.

^b^GEP: gender-expansive patient; percentages for GEP rows use the 366 GEP notes.

^c^NGEP: nongender-expansive patient; percentages for NGEP rows use the 388 NGEP notes.

^d^For GEP notes, the descriptor-excluding-misgendering variable retained notes containing nonmisgendering descriptors even when misgendering co-occurred. Among GEP notes, 51 contained nonmisgendering descriptors only, 51 contained both misgendering and a nonmisgendering descriptor, and 101 contained misgendering without a nonmisgendering descriptor.

Because misgendering constituted a large proportion of descriptor annotations, we also constructed an alternative descriptor variable excluding misgendering. Under this descriptor definition, 102 GEP notes contained nonmisgendering descriptor-related stigma. This included 51 notes with nonmisgendering descriptors only and 51 notes in which nonmisgendering descriptors co-occurred with misgendering. The descriptor variable excluding misgendering included 102 GEP notes. Section S5 of Multimedia Appendix 1 provides a schematic of the note-level overlap between nonmisgendering descriptors and misgendering. Correspondingly, the number of notes labeled as stigmatized decreased from 327 to 252 when misgendering was excluded from the descriptor category. This parallel representation of descriptor-related stigma allowed subsequent analyses to distinguish between disparities driven specifically by misgendering and those reflecting broader evaluative- or credibility-related language patterns.

**Figure 2:**
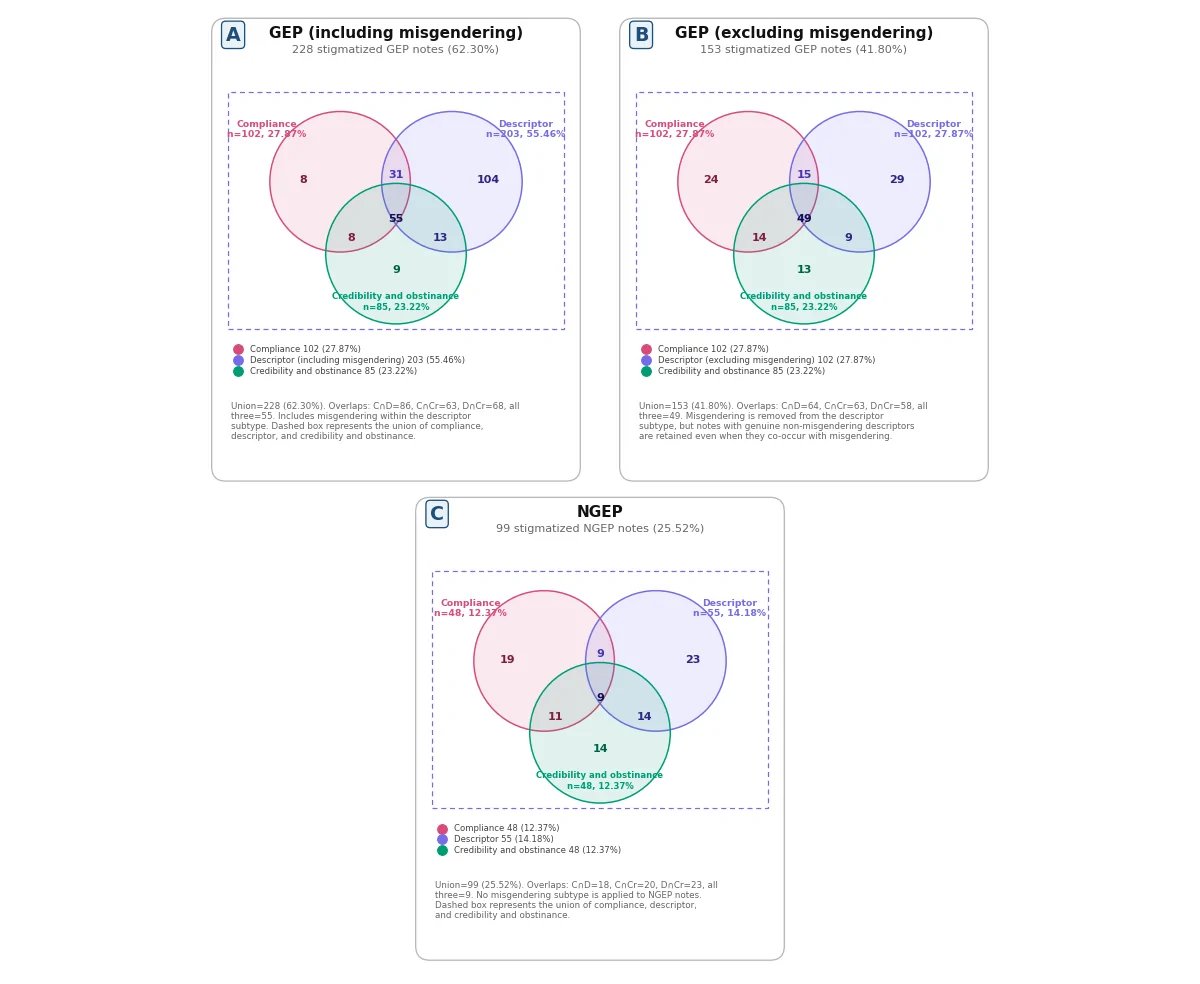
Overlap of stigmatizing language subtypes by group and misgendering definition. (A) Subtype overlaps among gender-expansive patient (GEP) notes when misgendering is included within the descriptor category. (B) Subtype overlap after excluding misgendering from the descriptor subtype while retaining co-occurring nonmisgendering descriptors. (C) Subtype overlaps among nongender-expansive patient (NGEP) notes. Numbers within each region indicate note counts, and percentages indicate the proportion of notes within the corresponding group.

Figure 2 visualizes the overlap structure underlying the subtype counts reported in Table 2. Among GEP notes, descriptor-related stigma was the most prevalent subtype when misgendering was included (203 notes), with substantial overlap across compliance-related and credibility- and obstinance-related stigma. After excluding misgendering from the descriptor category, descriptor-related stigma decreased to 102 notes; however, the note-level prevalence of SL remained elevated at 153 GEP notes, indicating that nonmisgendering descriptors, compliance-related language, and credibility- and obstinance-related language continued to contribute to stigmatizing documentation. In contrast, overlap patterns among NGEP notes were smaller in magnitude, consistent with the lower overall prevalence of SL observed in this group.

### Descriptive Patterns of SL Across Demographic Groups

As shown in Table 3, substantial disparities emerged between groups when stigma was defined as including misgendering. Overall stigma prevalence in GEP notes was 62.30% (228/366), more than twice that observed in NGEP notes (99/388, 25.52%). Within the NGEP group, SL appeared most frequently in documentation concerning Black/African American (26/54, 48.15%) and Asian (4/9, 44.44%) patients. Among GEPs, stigma prevalence was especially high for Hispanic/Latino (18/22, 81.82%) and Black/African American (27/43, 62.79%) patients.

Across language groups, stigma prevalence (including misgendering) was 24.51% (87/355) for NGEP English notes and 36.36% (12/33) for NGEP non-English notes. Among GEP notes, stigma prevalence was 62.85% (225/358) for English documentation and 37.50% (3/8) for non-English documentation. Across age quartiles, stigma prevalence among NGEP notes ranged from 24.56% to 29.55%. Among GEP notes, prevalence ranged from 56.90% to 69.86%, with the highest prevalence observed in the third quartile. These estimates should be interpreted cautiously because several demographic subgroups contained relatively small numbers of notes.

Overall, SL including misgendering appeared in approximately 2 of every 5 notes (327/754, 43.37%), with consistently higher prevalence among GEPs and racially minoritized patients. Descriptor-level stigma, particularly misgendering, emerged as the most frequent form of bias in the dataset. When misgendering was excluded from the descriptor category, overall stigma prevalence decreased to 33.42% (252/754) of notes. Under this alternative definition, stigma prevalence among GEP notes decreased from 62.30% (228/366) to 41.80% (153/366), whereas NGEP prevalence remained unchanged. These results indicate that although misgendering contributes substantially to descriptor-level stigma in GEP documentation, disparities in SL remain evident even when misgendering is excluded.

**Table 3:** Stigma prevalence by gender identity and demographic group.

Gender group and variable			Total notes, n		Stigmatized notes (with misgendering), n/N (%)		Stigmatized notes (excluding misgendering), n/N (%)
NGEP^a^							
	All notes	388		99/388 (25.52)		99/388 (25.52)	
	White	280		62/280 (22.14)		62/280 (22.14)	
	Black/African American	54		26/54 (48.15)		26/54 (48.15)	
	Hispanic/Latino	13		3/13 (23.08)		3/13 (23.08)	
	Asian	9		4/9 (44.44)		4/9 (44.44)	
	Race: other	32		4/32 (12.50)		4/32 (12.50)	
	English	355		87/355 (24.51)		87/355 (24.51)	
	Non-English	33		12/33 (36.36)		12/33 (36.36)	
	Age Q1 (18-29 years)	44		13/44 (29.55)		13/44 (29.55)	
	Age Q2 (30-46 years)	54		14/54 (25.93)		14/54 (25.93)	
	Age Q3 (47-62 years)	119		33/119 (27.73)		33/119 (27.73)	
	Age Q4 (63-91 years)	171		39/171 (22.81)		39/171 (22.81)	
GEP^b^							
	All notes	366		228/366 (62.30)		153/366 (41.80)	
	White	271		172/271 (63.47)		107/271 (39.48)	
	Black/African American	43		27/43 (62.79)		23/43 (53.49)	
	Hispanic/Latino	22		18/22 (81.82)		14/22 (63.64)	
	Asian	3		0/3 (0.00)		0/3 (0.00)	
	Race: other	27		11/27 (40.74)		9/27 (33.33)	
	English	358		225/358 (62.85)		151/358 (42.18)	
	Non-English	8		3/8 (37.50)		2/8 (25.00)	
	Age Q1 (18-29 years)	168		106/168 (63.10)		77/168 (45.83)	
	Age Q2 (30-46 years)	116		66/116 (56.90)		43/116 (37.07)	
	Age Q3 (47-62 years)	73		51/73 (69.86)		30/73 (41.10)	
	Age Q4 (63-91 years)	9		5/9 (55.56)		3/9 (33.33)	

^a^NGEP: nongender-expansive patient.

^b^GEP: gender-expansive patient.

### Multivariable Analysis of SL Across Demographic Groups

Multivariable logistic regression models were used to evaluate whether gender-expansive status remained associated with SL after adjusting for demographic covariates. When stigma was defined as including misgendering, GEP notes had substantially higher odds of containing SL compared with NGEP notes in the unadjusted model (OR 4.82, 95% CI 3.53-6.58; *P*<.001). This association remained stable after adjustment for race, primary language, and age (adjusted OR 4.87, 95% CI 3.54-6.70; *P*<.001), suggesting limited evidence of demographic confounding. Detailed regression results, interaction models, sensitivity analyses, and subtype-specific models are provided in Tables S3.1-S3.14 in section S3 of Multimedia Appendix 1.

Among covariates in the adjusted model, Black/African American race was independently associated with higher odds of stigmatizing documentation (OR 1.82, 95% CI 1.11-2.98; *P*=.02), whereas primary language and age group were not statistically significant predictors. The estimated OR for GEP status changed by less than 1% between the unadjusted and adjusted models, further supporting the robustness of the association. A sensitivity analysis excluding patients with race coded as Asian, Other, or Unknown yielded a slightly higher but consistent estimate (OR 5.60, 95% CI 3.97-7.91), indicating the robustness of the association. Model diagnostics suggested no meaningful multicollinearity, and the fully adjusted model showed adequate fit (Akaike information criterion [AIC]=927.61; McFadden pseudo-*R*^2^=0.1147).

To further evaluate the stability of the estimated disparity, repeated balanced subsampling analyses were conducted for the adjusted model described above. In each of 100 runs, notes were stratified by age group, race, and primary language, and within each subgroup, equal numbers of GEP and NGEP notes were randomly sampled using the minimum available count in that stratum. This procedure ensured comparable distributions across all demographic covariates included in the regression models while maintaining balance between groups within each subgroup. Logistic regression models were then estimated for each run using the same predictor structure as in the primary analysis, allowing us to assess whether the observed association persisted after balancing both group membership and demographic composition.

Across both balanced subsampling analyses, each of the 100 runs retained 614 notes total, with 307 GEP and 307 NGEP notes per run (mean total N=614.0, SD 0.0; range 614-614). Across runs, the balanced samples retained 17 matched demographic strata, all 4 age quartile levels (0 to 3), all 5 race levels (Asian, Black/African American, Hispanic/Latino, other, and White), and both language levels (English and non-English). Standard maximum-likelihood logistic regression failed to converge in some sparse, balanced samples. As prespecified, we used L1-regularized estimation as a fallback in 54 of 100 runs that included misgendering and in 62 of 100 runs that excluded misgendering. All runs produced valid estimates.

**Figure 3:**
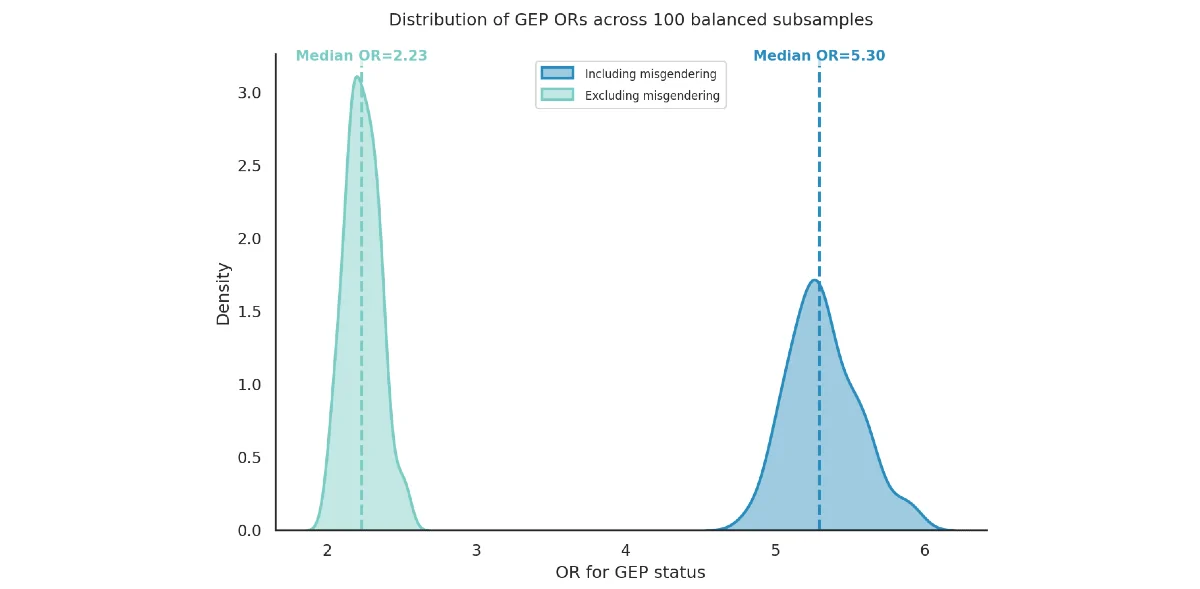
Stability of estimated disparities across repeated balanced subsamples. GEP: gender-expansive patient; OR: odds ratio.

Figure 3 shows the distribution of estimated ORs across these runs for models defined including and excluding misgendering. When misgendering was included, the estimated association between GEP status and SL remained consistently strong, with a mean OR of 5.32, an SD of 0.23, and a median of 5.30, with values ranging from 4.96 to 5.86 within the 95% CI. When misgendering was excluded, the magnitude of the association decreased but remained stable and statistically significant, with a mean OR of 2.24, an SD of 0.11, and a median of 2.23, with values ranging from 2.04 to 2.49 within the 95% CI. The narrow distributions across repeated subsamples indicate that the observed disparity is stable and not driven by a small subset of notes. In all subsamples, the association between GEP status and SL remained statistically significant (*P*<.05).

To evaluate whether this disparity was driven primarily by misgendering, parallel models were estimated using the alternative outcome definition excluding misgendering. Under this definition, GEP notes still had significantly higher odds of stigmatizing documentation in both the unadjusted (OR 2.10, 95% CI 1.54-2.86; *P*<.001) and adjusted models (adjusted OR 2.12, 95% CI 1.54-2.91; *P*<.001), although the magnitude of the association was attenuated relative to the primary model. In this alternative specification, Black/African American race remained associated with increased odds of SL (OR 2.33, 95% CI 1.46-3.72; *P*<.001), whereas primary language and age group were not significant predictors. A corresponding sensitivity analysis excluding patients with race coded as Asian, other, or unknown yielded a consistent estimate (OR 2.33, 95% CI 1.66-3.28). Model diagnostics were also acceptable for this specification (AIC=934.05; McFadden pseudo-*R*^2^=0.0424).

Exploratory interaction analyses assessed whether the association between GEP status and stigmatizing documentation varied across demographic groups. In these analyses, interaction models suggested 2 potential moderating patterns for the primary outcome. First, the disparity in stigma between GEPs and NGEPs was smaller among non-English speakers, although this estimate should be interpreted cautiously because of the limited size of the non-English GEP subgroup. Second, an exploratory GEP × Black/African American interaction suggested that, although Black patients exhibited higher baseline odds of stigmatizing documentation, the relative increase associated with GEP status appeared smaller among Black patients than among White patients (interaction OR 0.35, 95% CI 0.14-0.90; *P*=.03). No significant interaction was observed between GEP status and age group. These interaction findings are exploratory and should be interpreted cautiously given the limited subgroup sample sizes and reduced statistical power. Full regression outputs are presented in section S3 of Multimedia Appendix 1.

When misgendering was excluded, none of the interaction terms reached statistical significance. Specifically, no significant interactions were observed for GEP × language, GEP × age, or GEP × race. Consistent with this pattern, interaction models provided the most modest improvement over the baseline adjusted model. For the primary outcome including misgendering, the race-interaction model showed the best fit by AIC. For the alternative outcome excluding misgendering, interaction models produced only negligible improvement in model fit relative to the baseline specification.

These results indicate that although misgendering contributes substantially to the elevated prevalence of SL in GEP documentation, significant disparities remain even when misgendering is excluded, suggesting broader patterns of evaluative or credibility- and obstinacy-related bias in EHR notes.

### Subtype-Specific Multivariable Models of SL

To assess whether demographic disparities differed by linguistic form, separate multivariable models were estimated for each stigma subtype: credibility and obstinacy, compliance, and descriptors. Each model included GEP status as the primary predictor and adjusted for race, primary language, and age (section S3 of Multimedia Appendix 1). Across all subtypes, GEP status remained a significant and independent predictor of SL, although effect sizes varied by subtype. The association was strongest for descriptor-related stigma, followed by compliance-related stigma, and weakest for credibility- and obstinacy-related stigma.

When descriptors were defined as including misgendering, GEP notes had substantially higher odds of descriptor-related stigma compared with NGEP notes (OR 7.37, 95% CI 5.16-10.53; *P*<.001). Because misgendering constituted a large proportion of descriptor annotations in GEP documentation, an alternative model was estimated excluding misgendering from the descriptor category. Under this definition, the association between GEP status and descriptor-related stigma remained statistically significant but was substantially attenuated (OR 2.33, 95% CI 1.61-3.38; *P*<.001). For compliance-related stigma, GEP status was associated with increased odds of SL (OR 2.81, 95% CI 1.89-4.16; *P*<.001). For credibility- and obstinacy-related stigma, GEP notes also showed significantly higher odds compared with NGEP notes (OR 2.12, 95% CI 1.43-3.15; *P*<.001).

Demographic covariates also exhibited subtype-specific patterns. Black/African American race was associated with higher odds of both credibility- and obstinacy-related stigma (OR 2.14, 95% CI 1.26-3.64; *P*=.005) and compliance-related stigma (OR 2.61, 95% CI 1.57-4.34; *P*<.001), whereas older age groups were modestly less likely to be described using compliance-related SL (OR 0.84, 95% CI 0.70-0.99; *P*=.04). Primary language was not a significant predictor in any subtype model. All models converged successfully, with no evidence of multicollinearity or instability.

A separate regression model was estimated for misgendering as an outcome. However, because misgendering was observed almost exclusively in GEP notes, the model exhibited near-complete separation and produced unstable parameter estimates. As a result, misgendering was treated primarily as a descriptive subtype rather than as a comparative regression outcome. Overall, these subtype-specific analyses indicate that stigmatizing documentation associated with gender-expansive status manifests across multiple linguistic forms, including credibility and obstinacy framing, compliance judgments, and evaluative descriptors. Although misgendering represents a prominent component of descriptor-related stigma, significant disparities persist across other stigma subtypes as well.

### Cross-Domain Generalization of MSTL Models

**Figure 4:**
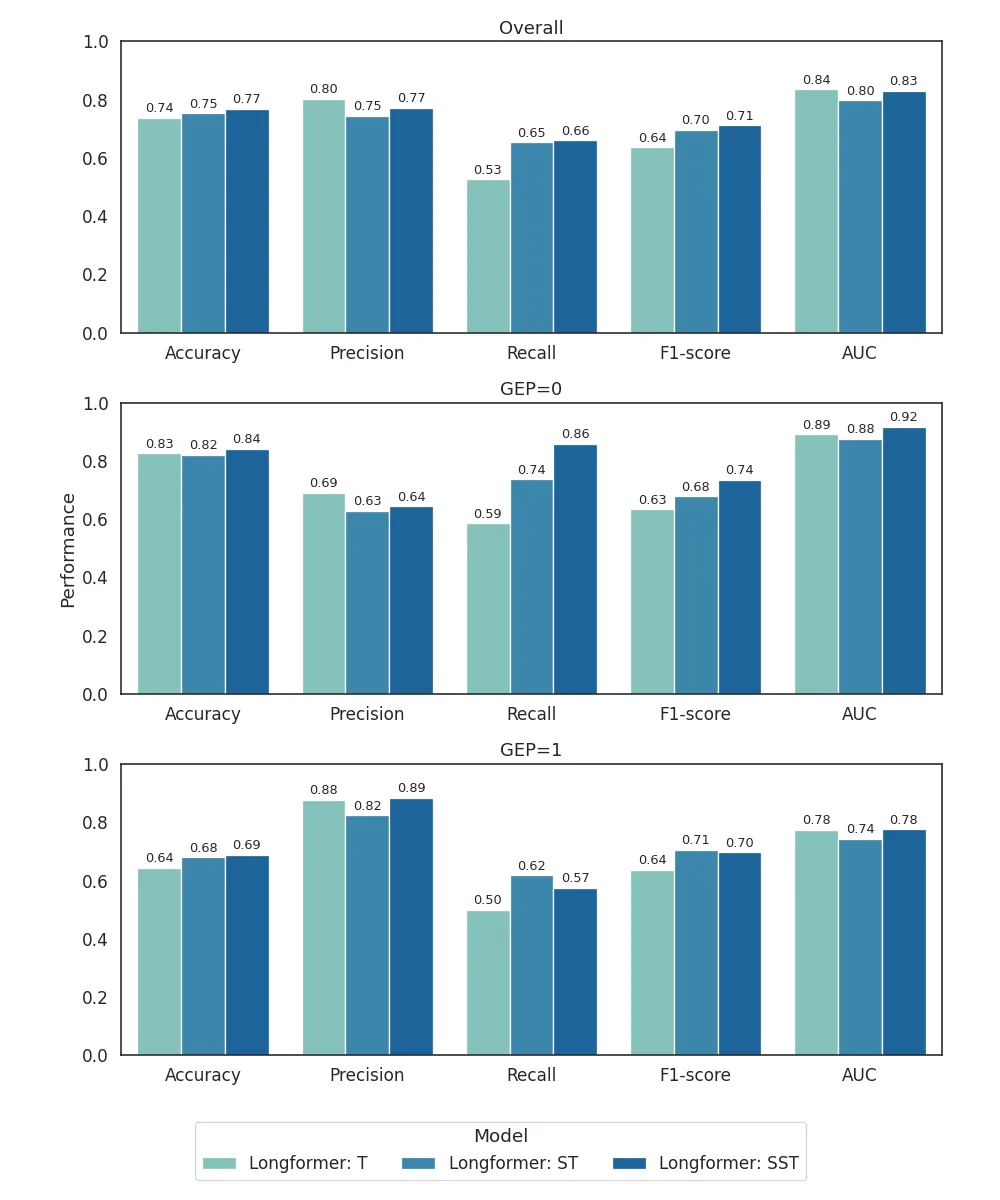
Cross-domain performance of multistage transfer learning models by group including misgendering (gender-expansive patient [GEP]=0 indicates the nongender-expansive patient [NGEP] group; GEP=1 indicates the GEP group). AUC: area under the curve; SST: semantic, syntactic, and task-based fine-tuning; ST: syntactic and task-based fine-tuning; T: task-based fine-tuning.

We evaluated the cross-domain performance of previously developed MSTL Longformer models by applying them directly to the newly constructed GEP dataset without additional fine-tuning [20]. This experiment assessed whether linguistic representations learned from prior training stages generalized to gender-expansive clinical documentation. Each stage of the MSTL framework was applied directly to the GEP dataset without additional fine-tuning. As shown in Figure 4, MSTL models achieved moderate overall performance, with the fully adapted Longformer model with semantic, syntactic, and task-based fine-tuning (SST) performing best. While discrimination was strong for NGEP notes, performance declined for GEP notes, primarily due to reduced recall. These results indicate that stigma-related linguistic patterns learned from prior source domains transfer less effectively to GEP-related documentation.

**Figure 5:**
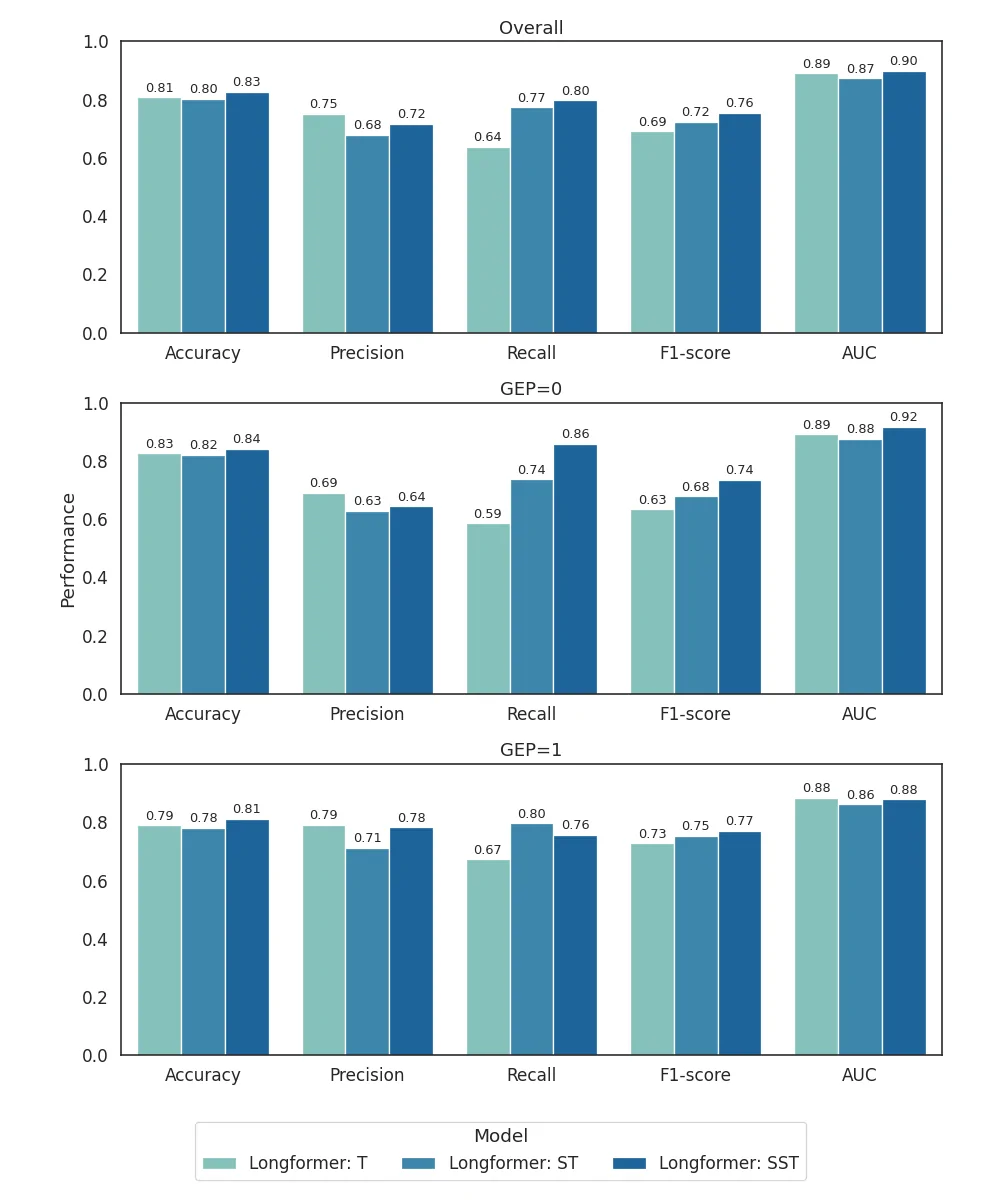
Cross-domain performance of multistage transfer learning models by group excluding misgendering (gender-expansive patient [GEP]=0 indicates the nongender-expansive patient [NGEP] group). AUC: area under the curve; SST: semantic, syntactic, and task-based fine-tuning; ST: syntactic and task-based fine-tuning; T: task-based fine-tuning.

To examine the role of misgendering in model behavior, the same cross-domain evaluation was repeated using the alternative outcome definition excluding misgendering. As shown in Figure 5, overall model performance improved under this definition. Although subgroup differences remained, the disparity between GEP and NGEP notes was smaller relative to the primary analysis, suggesting that misgendering contributes substantially to cross-domain detection difficulty.

### Benchmark Performance of Models Trained on the GEP Dataset

To benchmark SL detection when models were trained directly on the GEP dataset, we evaluated a broader set of models, including Longformer variants with GEP-based fine-tuning, BERT, ClinicalBERT, and several traditional machine learning classifiers. Model performance on the held-out testing set is summarized in Table 4. PR-AUC values are also reported to provide a more informative evaluation under class imbalance, particularly for low-prevalence stigma subtypes. Because PR-AUC is prevalence dependent, subgroup PR-AUC values were interpreted relative to subgroup-specific positive-class prevalence. Given the higher baseline prevalence of SL among GEP notes than among NGEP notes, comparable raw PR-AUC values across groups may correspond to smaller gains over baseline in the GEP subgroup. Across models, PR-AUC values were generally lower than ROC-AUC values, except in subgroups in which the positive class was the majority. In the GEP subgroup, for which the positive-class prevalence was 62.30%, PR-AUC frequently exceeded ROC-AUC. Overall, the 2 metrics supported similar performance trends across models.

Among the evaluated models, Longformer architectures incorporating staged transfer learning achieved the strongest overall performance. Longformer: SST + GEP, which applies additional task-specific fine-tuning on the GEP/NGEP dataset introduced in this study, achieved the best overall performance when SL was defined as including misgendering (accuracy=0.8278; *F*_1_-score=0.8000; ROC-AUC=0.8809; PR-AUC=0.8475). When misgendering was excluded, the same model achieved improved results (accuracy=0.8808; *F*_1_-score=0.8200; ROC-AUC=0.9229; PR-AUC=0.8074).

Across models, performance differed between NGEP and GEP subsets. In several cases, NGEP notes achieved higher accuracy and AUC, whereas GEP notes sometimes exhibited comparable or higher precision or recall, depending on the architecture. Traditional machine learning models generally showed weaker performance relative to transformer-based models, although they demonstrated similar subgroup performance patterns. These results indicate that while domain-adapted transformer models substantially improve overall detection of SL, performance differences across patient groups remain evident.

Model performance was also evaluated separately for each stigma subtype, and the subtype-specific benchmarking results are reported in section S4 of Multimedia Appendix 1. Briefly, the Longformer: SST achieved the strongest performance for linguistically nuanced subtypes such as credibility and obstinacy and descriptors (including misgendering in this analysis), whereas the standard Longformer performed best for compliance, and ClinicalBERT achieved the highest accuracy for misgendering. Across subtypes, the best-performing models achieved ROC-AUC values above 0.73, indicating stable discrimination despite substantial class imbalance. Complete model evaluation results are provided in Multimedia Appendix 2.

**Table 4:** Model performance across the overall, GEP^a^, and NGEP^b^ subsets (testing set). GEP=1 indicates GEP notes; GEP=0 indicates NGEP notes.

Model and group				Accuracy		Precision		Recall		*F*_1_-score		ROC-AUC^c^		PR-AUC^d^
Transformer models														
	Longformer: GEP													
		Overall	0.7285		0.6579		0.7692		0.7092		0.8163		0.7763	
		GEP=0	0.7722		0.5385		0.7000		0.6087		0.8322		0.7023	
		GEP=1	0.6806		0.7200		0.8000		0.7579		0.7045		0.8063	
	Longformer: GEP (excluding misgendering)													
		Overall	0.8013		0.6780		0.7843		0.7273		0.8506		0.7680	
		GEP=0	0.8354		0.6296		0.8500		0.7234		0.8508		0.7052	
		GEP=1	0.7639		0.7188		0.7419		0.7302		0.8395		0.8083	
	Longformer: T^e^ + GEP													
		Overall	0.8079		0.7813		0.7692		0.7752		0.8150		0.7498	
		GEP=0	0.8734		0.6923		0.9000		0.7826		0.8839		0.7044	
		GEP=1	0.7361		0.8421		0.7111		0.7711		0.7432		0.8158	
	Longformer: T + GEP (excluding misgendering)													
		Overall	0.8609		0.7885		0.8039		0.7961		0.8973		0.8196	
		GEP=0	0.9367		0.8261		0.9500		0.8837		0.9492		0.8359	
		GEP=1	0.7778		0.7586		0.7097		0.7333		0.8363		0.7979	
	Longformer: ST^f^ + GEP													
		Overall	0.8146		0.7937		0.7692		0.7813		0.8526		0.7987	
		GEP=0	0.8734		0.7083		0.8500		0.7727		0.9203		0.7676	
		GEP=1	0.7500		0.8462		0.7333		0.7857		0.7786		0.8607	
	Longformer: ST + GEP (excluding misgendering)													
		Overall	0.8676		0.7541		0.9020		0.8214		0.9227		0.8294	
		GEP=0	0.8861		0.7391		0.8500		0.7907		0.9237		0.7293	
		GEP=1	0.8472		0.7632		0.9355		0.8406		0.9284		0.9116	
	Longformer: SST^g^ + GEP													
		Overall	0.8278		0.8000		0.8000		0.8000		0.8809		0.8475	
		GEP=0	0.8861		0.7391		0.8500		0.7907		0.9153		0.8527	
		GEP=1	0.7639		0.8333		0.7778		0.8046		0.8025		0.8542	
	Longformer: SST + GEP (excluding misgendering)													
		Overall	0.8808		0.8367		0.8039		0.8200		0.9229		0.8074	
		GEP=0	0.8987		0.8000		0.8000		0.8000		0.9322		0.8481	
		GEP=1	0.8611		0.8621		0.8065		0.8333		0.8969		0.7991	
	BERT^h^													
		Overall	0.7748		0.7246		0.7692		0.7463		0.8093		0.7535	
		GEP=0	0.8481		0.7222		0.6500		0.6842		0.7966		0.7163	
		GEP=1	0.6944		0.7255		0.8222		0.7708		0.6897		0.7715	
	BERT (excluding misgendering)													
		Overall	0.8079		0.8235		0.5490		0.6588		0.7927		0.7150	
		GEP=0	0.8481		1.0000		0.4000		0.5714		0.7237		0.6459	
		GEP=1	0.7639		0.7692		0.6452		0.7018		0.8293		0.7868	
	ClinicalBERT													
		Overall	0.7417		0.6806		0.7538		0.7153		0.8125		0.7527	
		GEP=0	0.7722		0.5357		0.7500		0.6250		0.8220		0.6780	
		GEP=1	0.7083		0.7727		0.7556		0.7640		0.7251		0.7831	
	ClinicalBERT (excluding misgendering)													
		Overall	0.7815		0.6957		0.6275		0.6598		0.8218		0.7158	
		GEP=0	0.7975		0.6429		0.4500		0.5294		0.7432		0.6303	
		GEP=1	0.7639		0.7188		0.7419		0.7302		0.8363		0.7617	
Traditional models (grid-search optimized)														
	SVM^i^													
		Overall	0.781457		0.7667		0.7077		0.7360		0.7882		0.7630	
		GEP=0	0.7848		0.5882		0.5000		0.5405		0.7017		0.5881	
		GEP=1	0.7778		0.8372		0.8000		0.8182		0.7704		0.8335	
	SVM (excluding misgendering)													
		Overall	0.7748		0.6809		0.6275		0.6531		0.7918		0.6915	
		GEP=0	0.7468		0.5000		0.4000		0.4444		0.6475		0.5065	
		GEP=1	0.8056		0.7742		0.7742		0.7742		0.8592		0.7883	
	RF^j^													
		Overall	0.7483		0.8000		0.5538		0.6545		0.8199		0.7775	
		GEP=0	0.7975		0.7000		0.3500		0.4667		0.8000		0.6504	
		GEP=1	0.6944		0.8286		0.6444		0.7250		0.7654		0.8222	
	RF (excluding misgendering)													
		Overall	0.8013		0.8621		0.4902		0.6250		0.8441		0.7722	
		GEP=0	0.7975		1.0000		0.2000		0.3333		0.7788		0.6668	
		GEP=1	0.8056		0.8400		0.6774		0.7500		0.8749		0.8287	
	LR^k^													
		Overall	0.7748		0.7541		0.7077		0.7302		0.7918		0.7662	
		GEP=0	0.7722		0.5556		0.5000		0.5263		0.7017		0.5887	
		GEP=1	0.7778		0.8372		0.8000		0.8182		0.7728		0.8354	
	LR (excluding misgendering)													
		Overall	0.7748		0.6809		0.6275		0.6531		0.7935		0.7022	
		GEP=0	0.759494		0.5333		0.4000		0.4571		0.6475		0.5150	
		GEP=1	0.7917		0.7500		0.7742		0.7619		0.8544		0.7935	
	NB^l^													
		Overall	0.728477		0.7609		0.5385		0.6306		0.7408		0.7279	
		GEP=0	0.7595		0.5455		0.3000		0.3871		0.6415		0.5301	
		GEP=1	0.6944		0.8286		0.6444		0.7250		0.7407		0.8191	
	NB (excluding misgendering)													
		Overall	0.7881		0.7317		0.5882		0.6522		0.7782		0.6995	
		GEP=0	0.7975		0.7500		0.3000		0.4286		0.6492		0.5491	
		GEP=1	0.7778		0.7273		0.7742		0.7500		0.8293		0.7750	

^a^GEP: gender-expansive patient.

^b^NGEP: nongender-expansive patient.

^c^ROC-AUC: area under the receiver operating characteristic curve.

^d^PR-AUC: area under the precision-recall curve.

^e^T: task-based fine-tuning.

^f^ST: syntactic and task-based fine-tuning.

^g^SST: semantic, syntactic, and task-based fine-tuning.

^h^BERT: Bidirectional Encoder Representations from Transformers.

^i^SVM: support vector machine.

^j^RF: random forest.

^k^LR: logistic regression.

^l^NB: naive Bayes.

### Comparative Performance and Fairness Assessment Under Baseline and Equalized Odds-Constrained Thresholding

**Figure 6:**
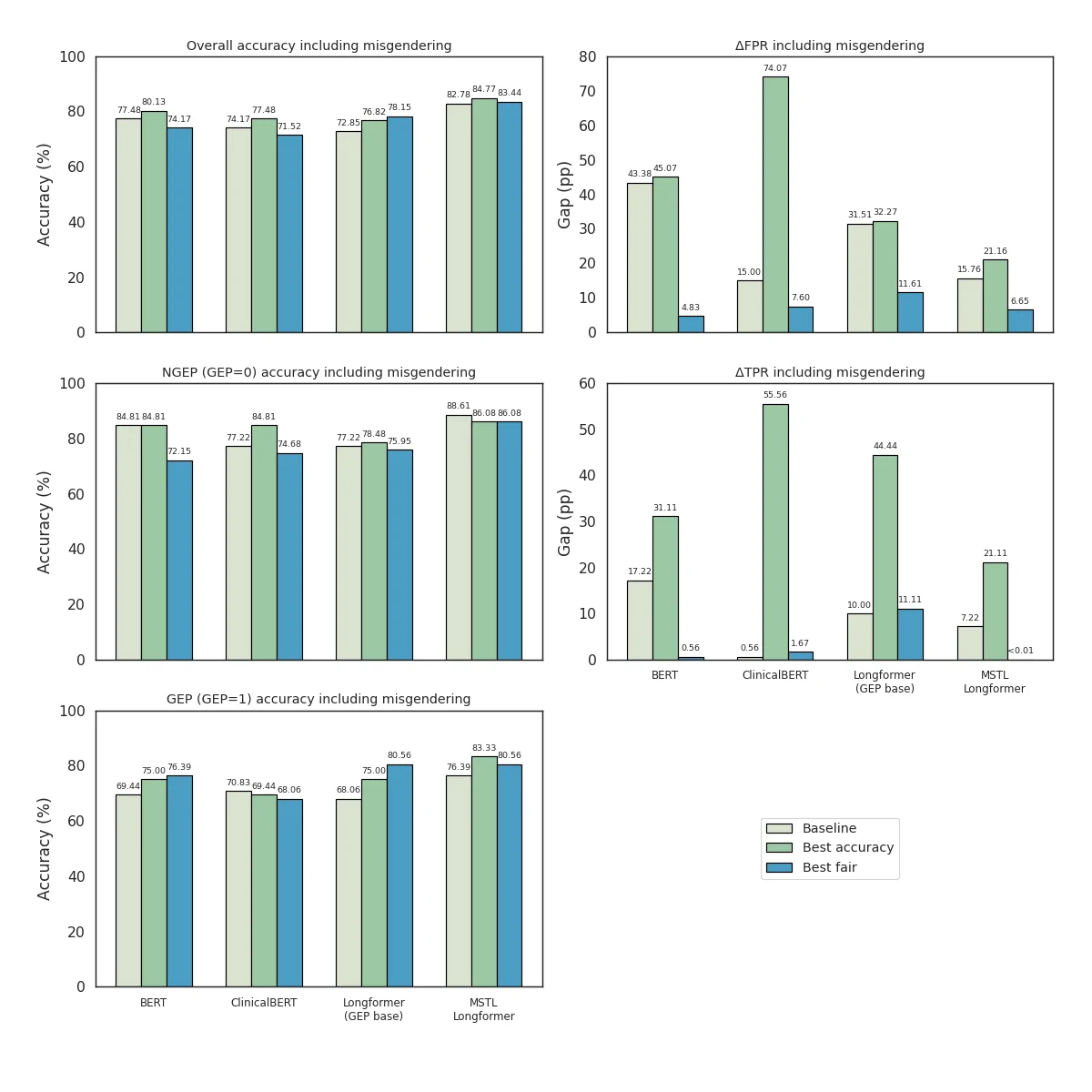
Accuracy and subgroup fairness under baseline and threshold-optimized configurations including misgendering. BERT: Bidirectional Encoder Representations from Transformers; FPR: false-positive rate; GEP: gender-expansive patient; MSTL: multistage transfer learning; NGEP: nongender-expansive patient; pp: percentage points; TPR: true-positive rate.

**Figure 7:**
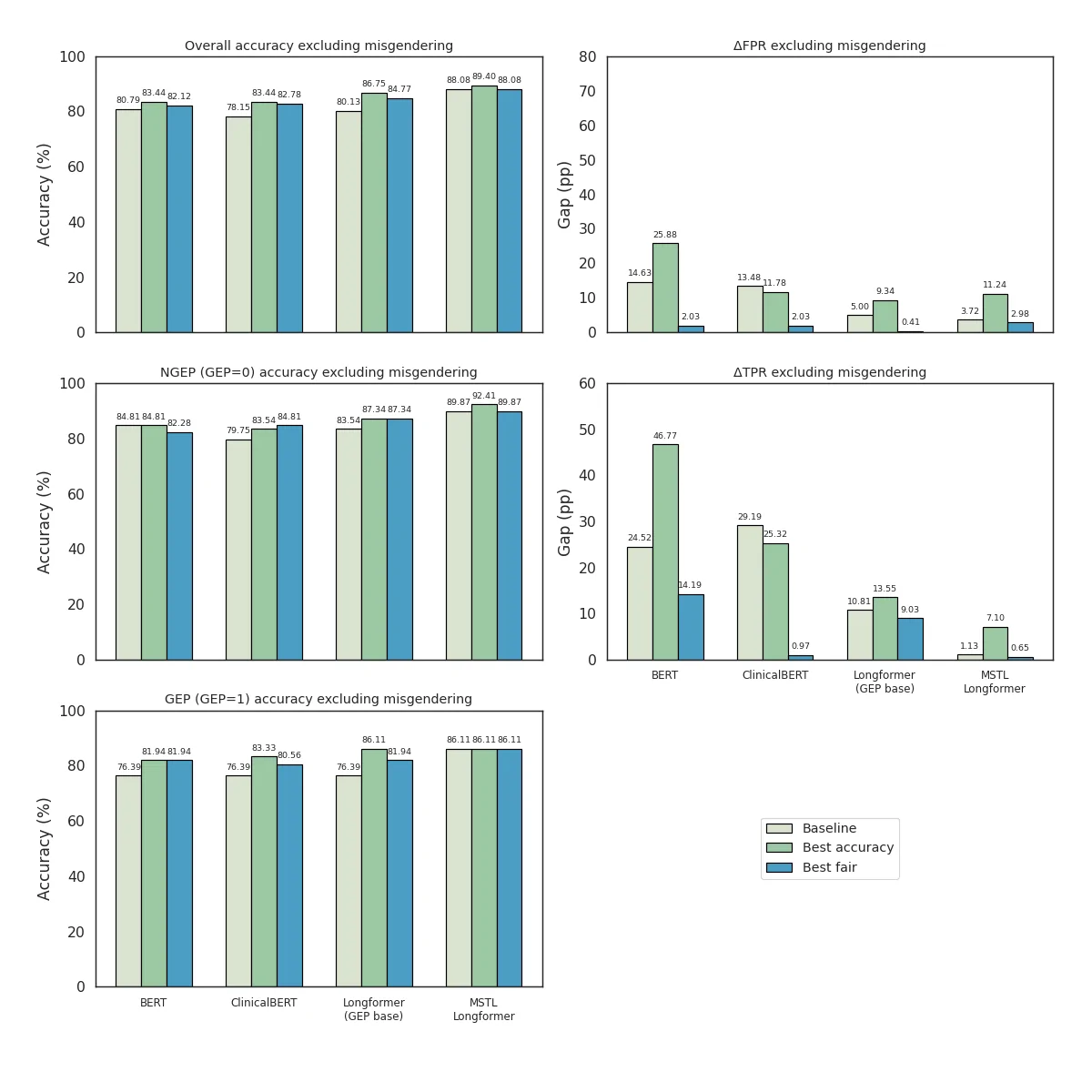
Accuracy and subgroup fairness under baseline and threshold-optimized configurations excluding misgendering. BERT: Bidirectional Encoder Representations from Transformers; FPR: false-positive rate; GEP: gender-expansive patient; MSTL: multistage transfer learning; NGEP: nongender-expansive patient; pp: percentage points; TPR: true-positive rate.

We evaluated subgroup performance for 4 transformer-based models under 3 decision-threshold configurations: baseline (global threshold=0.5), best-accuracy, and best-fair, using post hoc equalized odds-constrained threshold optimization. Performance was assessed separately for 2 outcome definitions: stigma including misgendering and stigma excluding misgendering. Accuracy for the overall test set and for each subgroup (GEP=1; NGEP=0), as well as subgroup fairness metrics (ΔFPR and ΔTPR), are shown in Figures 6 and 7.

Under the baseline threshold, overall accuracy ranged from 72.85% (110/151) for the Longformer base model to 82.78% (125/151) for the MSTL-Longformer (Figure 6). However, substantial subgroup disparities were observed. For example, BERT achieved 84.81% (67/79) accuracy for NGEP notes but only 69.44% (50/72) for GEP notes, whereas the Longformer base model achieved 77.22% (61/79) for NGEP notes and 68.06% (49/72) for GEP notes. Correspondingly, fairness gaps were large, with ΔFPR reaching 43.38 percentage points (pp) for BERT and 31.51 pp for the Longformer base model. The MSTL-Longformer exhibited the most balanced baseline behavior, with ΔFPR=15.76 pp and ΔTPR=7.22 pp while maintaining the highest overall accuracy.

Applying threshold optimization produced the expected trade-off between accuracy and fairness. The best-accuracy configuration increased overall performance but generally widened subgroup disparities. For example, BERT achieved 80.13% (121/151) overall accuracy under the best-accuracy threshold, but ΔFPR increased to 45.07 pp and ΔTPR to 31.11 pp. In contrast, the best-fair configuration substantially reduced subgroup differences across all models. For the MSTL-Longformer, ΔFPR decreased from 15.76 pp to 6.65 pp and ΔTPR from 7.22 pp to near-zero levels, with only minimal change in overall accuracy (126/151, 83.44% vs 125/151, 82.78%). However, these fairness estimates should be interpreted cautiously because subgroup-specific metrics are based on relatively small numbers of positive instances and may be sensitive to individual predictions. Similar patterns were observed for BERT and ClinicalBERT, although these models experienced larger accuracy reductions when fairness constraints were applied. These findings highlight that fairness improvements in error rates may be accompanied by differences in the reliability of positive predictions across groups.

We additionally examined group-specific precision (positive predictive value) across models. Precision differed between groups, reflecting the expected trade-off under unequal baseline prevalence. For example, in the MSTL-Longformer model (including misgendering), precision was 0.74 for the NGEP group and 0.83 for the GEP group. Similar patterns were observed across models and outcome definitions. Detailed group-specific performance metrics are provided in Table S6 in Section S6 of Multimedia Appendix 1.

When misgendering was excluded from the outcome definition, overall performance increased for all models, and subgroup disparities were generally smaller at baseline (Figure 7). Baseline overall accuracy ranged from 78.15% (118/151) for ClinicalBERT to 88.08% (133/151) for the MSTL-Longformer. For example, the Longformer base model achieved 83.54% (66/79) accuracy for NGEP notes and 76.39% (55/72) for GEP notes, with ΔFPR=5.00 pp and ΔTPR=10.81 pp. The MSTL-Longformer again showed the strongest baseline performance, with overall accuracy of 88.08% (133/151), ΔFPR=3.72 pp, and ΔTPR=1.13 pp.

Threshold optimization again revealed the accuracy-fairness trade-off. The best-accuracy configuration produced the highest overall accuracy but increased subgroup disparities, whereas the best-fair configuration consistently reduced ΔFPR across all models. For example, under fairness-optimized thresholding, ΔFPR for the Longformer base model decreased from 5.00 pp to 0.41 pp, and ΔFPR for ClinicalBERT decreased from 13.48 pp to 2.03 pp. For the MSTL-Longformer, fairness optimization reduced ΔFPR to 2.98 pp and ΔTPR to 0.65 pp while maintaining high overall accuracy (133/151, 88.08%). These results indicate that excluding misgendering reduces both overall difficulty and subgroup imbalance, but fairness constraints remain necessary to minimize residual disparities.

Across both outcome definitions, the MSTL-Longformer consistently provided the best balance between predictive performance and subgroup fairness. Models trained without staged transfer learning exhibited larger subgroup gaps and required stronger threshold adjustment to achieve comparable fairness. Importantly, fairness optimization reduced subgroup disparities without retraining, but in most cases, this came at the cost of reduced overall accuracy, particularly for BERT and ClinicalBERT. These findings demonstrate that subgroup fairness in stigmatizing-language detection depends not only on model architecture but also on the definition of stigma and the decision threshold used for classification.

## Discussion

### Principal Findings

This study provides quantitative evidence that SL is common in EHR documentation and disproportionately affects GEPs. The high prevalence of SL, the dominance of misgendering within descriptor-level stigma, and the persistence of these patterns after demographic adjustment suggest consistent disparities in clinical documentation. In analyses using 2 outcome definitions, one including misgendering and one excluding misgendering, misgendering accounted for a substantial portion of descriptor-level stigma but did not fully explain the elevated prevalence of SL among GEP notes. The persistence of disparities after excluding misgendering suggests that stigmatizing documentation extends beyond incorrect gender references to broader evaluative, credibility- and obstinacy-related, and compliance-related framing.

Misgendering represents an explicit lexical error, whereas other forms of SL are often conveyed through tone, narrative emphasis, or credibility-undermining phrasing. Separating outcome definitions therefore allows a more precise characterization of SL in clinical documentation. At the same time, the note-level annotation strategy used in this study has important implications for how these results should be interpreted. Although downstream clinical outcomes were not directly examined, prior work shows that SL in medical records shapes how subsequent clinicians interpret patients and engage in care [9]. Broader reviews similarly indicate that credibility-undermining and stigmatizing phrasing varies by race, suggesting that EHRs reflect and reinforce structural inequities [37], and that clinical text is a key site where inequity may be algorithmically amplified [38].

Note-level labeling also has important implications for model learning and interpretation. Because labels indicate the presence of any SL within a note, models may rely more on global contextual cues rather than localized linguistic spans, in contrast to span-level supervision approaches. Additionally, fairness metrics derived from note-level labels may be influenced by note length and label granularity, and prevalence estimates should be interpreted as reflecting “any-instance presence” rather than the overall tone or density of SL within a note. As a result, prevalence estimates may differ from span-level approaches, potentially overestimating the presence of stigma at the note level while underrepresenting its frequency or intensity within notes.

Interpreting the observed GEP × race interaction requires an intersectional perspective. Interaction terms were included to explore whether the association between gender-expansive status and SL varied across demographic groups, consistent with prior literature showing that clinical documentation bias and health disparities may differ across intersecting identities [8,32,33]. Because GEPs may experience overlapping forms of marginalization, examining interaction effects provides a way to evaluate whether disparities operate differently across subgroups rather than assuming a uniform effect. The observed attenuation of the incremental effect of GEP status among Black patients in exploratory analyses is broadly consistent with prior work showing that racialized documentation patterns can influence how clinicians describe patients, often resulting in increased use of judgmental or credibility-related language regardless of other characteristics [39,40]. Such a “saturation effect” may reduce the detectable incremental influence of gender identity in EHRs.

At the same time, transgender and gender-expansive identities may be underrecognized or underdocumented among racial minority groups, consistent with evidence that transgender people of color face heightened invisibility and misrecognition in clinical care [3,41]. These mechanisms offer plausible explanations for why interaction effects appear attenuated in exploratory analyses even when both racial minority status and gender-expansive identity independently exhibit high stigma exposure. However, several interaction strata contained small numbers of notes, including non-English-speaking GEP notes and certain racial subgroups. These small sample sizes limit statistical power for interaction testing and may produce unstable estimates. Therefore, the interaction results should be interpreted as exploratory and hypothesis-generating rather than as definitive evidence of subgroup-specific differences.

During the annotation process, we noted that some EHR notes contained multiple stigmatizing descriptors within the same encounter, particularly in notes describing Black patients, where behavioral, substance-related, or risk-focused language sometimes appeared together (eg, references to agitation, nonadherence, substance use, or social instability). In such cases, stigmatizing framing was already extensive, which may reduce the measurable incremental effect of gender identity in the regression models used in this study. This overlap of racialized and behavior-focused documentation patterns may partially explain why interaction terms for gender identity and race did not show additive increases in risk despite elevated baseline stigma. Hence, this overlap would seem to be an area ripe for exploration with formal qualitative analysis in future work.

Because EHR notes are reused across encounters and increasingly analyzed at scale, SL can influence care beyond a single visit. Such language shapes future clinicians’ interpretations and may contribute to cumulative bias over time [9,15]. It also affects secondary uses of clinical text, where disproportionate negative descriptors can distort research datasets, quality metrics, and health-system analytics [4,40]. As large language models and other AI systems increasingly train on EHR data, documentation bias risks becoming embedded in downstream clinical NLP and decision-support tools, potentially reinforcing inequities affecting GEPs and Black patients [42,43]. Expanded patient access to notes through OpenNotes further underscores the importance of affirming documentation, as patients report that SL undermines trust while respectful documentation strengthens engagement [44,45].

In practical deployment, such models may be integrated into clinical documentation workflows as auditing or quality-improvement tools that flag potential instances of SL in EHR notes for review. Rather than serving as automated decision-makers, these systems are best positioned as assistive tools that support clinician awareness and documentation refinement. Effective implementation would require ongoing monitoring of both overall performance and subgroup fairness metrics to ensure that disparities do not emerge or worsen over time. In addition, model outputs should be interpreted as probabilistic signals rather than definitive judgments and should be used in conjunction with human review to support appropriate and context-sensitive interventions. Recent work on clinical language-based AI systems has similarly emphasized that responsible deployment requires careful evaluation of interpretability, reliability, and subgroup performance rather than reliance on aggregate performance metrics alone [46]. These considerations are particularly important in clinical environments, where language-based AI systems may influence quality monitoring, documentation review, or downstream analytic workflows involving sensitive patient populations.

Within this context, our modeling results highlight both promise and limitations. The MSTL framework improved detection performance relative to traditional baselines by enabling capture of contextual nuance and cross-sentence tone characteristic of stigmatizing documentation [7,9]. This advantage is particularly important for long EHR notes, where SL is often expressed through multisentence context, narrative framing, or subtle evaluative wording rather than isolated keywords. MSTL therefore shows potential as a screening or quality assurance approach for identifying misgendering and other bias-linked expressions in EHR workflows. The present analysis focused on transformer architectures commonly used in clinical NLP research rather than large generative language models. Although large language models may offer improved contextual understanding, their use in protected clinical text introduces additional challenges related to computational cost, reproducibility, and privacy constraints. Evaluation of such models remains an important direction for future work.

Persistent performance gaps for GEP-related text indicate that high accuracy alone does not ensure equitable detection. Performance also differed depending on the definition of stigma. When misgendering was included, subgroup disparities were larger, whereas excluding misgendering reduced overall difficulty but did not eliminate performance gaps, suggesting that models struggle with more implicit forms of SL. Models trained primarily on majority group narratives may underidentify stigma in marginalized populations unless representational diversity and fairness-sensitive strategies are intentionally incorporated, consistent with broader evidence on bias inheritance in clinical AI systems [42,43]. Unlike prior work focused on general SL detection, the present study evaluates performance, subgroup disparities, and fairness optimization within a gender-expansive-inclusive corpus, providing a more detailed assessment of bias in clinical NLP systems.

Our fairness analysis shows that even high-performing transformer models exhibit substantial disparities in false-positive and false-negative rates for GEP documentation. Default thresholds often produce large gaps between GEP and NGEP groups, highlighting how standard deployment can disproportionately affect marginalized patients. These disparities were observed under both outcome definitions, although they were larger when misgendering was included. Notably, higher overall accuracy did not guarantee equitable subgroup performance, underscoring the limits of single-metric evaluation. In clinical quality-monitoring settings, false positives may incorrectly label neutral documentation as stigmatizing, while false negatives allow harmful language to persist. In this context, subgroup fairness metrics provide information relevant to real-world deployment decisions that cannot be captured by accuracy alone. In clinical auditing workflows, however, reducing subgroup disparities should not come at the cost of missing severe instances of SL, because excessive false negatives could undermine the system’s intended safety and quality-improvement function. Fairness metrics such as ΔFPR and ΔTPR revealed disparities not captured by accuracy alone, highlighting trade-offs between false positives and false negatives.

When comparing constrained and unconstrained threshold strategies, we demonstrate that meaningful fairness gains are achievable without sacrificing clinically relevant accuracy. The fairness evaluation in this study focused primarily on disparities between GEP and NGEP groups because this was the primary objective of the analysis. Intersectional disparities across race, language, and other demographic factors were not fully evaluated and are discussed as a limitation below. Among the models tested, the MSTL-Longformer showed the most consistent balance between performance and subgroup fairness across threshold settings. Post hoc thresholding offers a practical, model-agnostic intervention when retraining large models is not feasible. However, this approach requires knowledge of group membership at inference time. In practice, gender identity fields in EHRs may be incomplete or inconsistently recorded, particularly for GEPs. This limitation highlights a deployment challenge for fairness-aware systems, as interventions designed to reduce bias may depend on attributes that are themselves imperfectly documented. This creates a practical tension for real-world deployment because fairness interventions that rely on accurate demographic labels may be difficult to apply when those labels are missing or unreliable. Therefore, the threshold-based fairness strategy demonstrated here should be interpreted as a proof-of-concept approach, and future work will be needed to develop mitigation methods that do not require protected attributes at inference time. These findings emphasize embedding fairness auditing throughout the clinical NLP lifecycle as a core principle of responsible AI.

### Limitations and Future Directions

Several limitations should be considered when interpreting these findings. While stigma in health care has been documented, large-scale quantitative analyses of SL toward GEPs remain rare, and this study represents an early step toward building systematic evidence in this area. As such, several design decisions reflect trade-offs required to study relatively uncommon linguistic phenomena.

The dataset was constructed using targeted keyword filtering to ensure sufficient representation of SL. Although necessary for reliable annotation and modeling, this enrichment may inflate prevalence estimates relative to untargeted samples by preferentially capturing notes in which gender identity is already salient. At the same time, this approach may underrepresent GEP patients whose gender identity is not documented, thereby limiting generalizability. As a result, both prevalence estimates and effect sizes (eg, ORs) may be inflated relative to the broader patient population. In addition, the keyword-based strategy used to identify GEPs has been used in prior work but was not validated against self-reported gender identity within MIMIC-IV and may preferentially capture notes in which identities were explicitly documented while missing patients whose gender identity was recorded using other terms [29-31]. Consequently, prevalence estimates should be interpreted as comparative rates within an enriched corpus rather than population-level estimates of stigma. Nevertheless, the presence of gender-related keywords in reclassified NGEP notes may provide salient lexical cues and could influence model behavior; future work should evaluate sensitivity to excluding or separately modeling these keyword-bearing controls.

The data originate from a single institution and historical period and primarily reflect inpatient documentation in an academic medical center, which may limit generalizability to other clinical settings. Although the NGEP cohort was sampled to approximate the marginal distributions of race, age, and primary language observed among GEPs, exact demographic parity was not achieved across all strata. Some subgroup distributions remained imbalanced, which may affect subgroup prevalence estimates and interaction analyses. Fairness evaluation focused primarily on disparities between GEP and NGEP groups, and limited sample sizes in several intersectional strata reduced the stability of multidimensional fairness metrics.

Gender-affirming documentation norms have evolved over time, and linguistic patterns observed in notes from 2008 to 2019 may not fully reflect current practices. In addition, structured gender fields in EHRs often reflect sex assigned at birth rather than affirmed gender identity and may be inconsistent for GEPs, particularly in historical datasets. Because of these limitations, gender was not used as a matching variable or regression covariate, which prevented evaluation of documentation differences across cisgender male and female patients. The annotated gender-expansive corpus also remains small for some subgroups, limiting statistical power and generalizability across diverse gender identities, race categories, and clinical contexts. Interaction analyses involving race and language should therefore be interpreted as exploratory. Age quartile was modeled ordinally to preserve degrees of freedom in a relatively small analytic sample. This specification assumes an approximately monotonic change in log-odds across adjacent age brackets and may not capture nonlinear or nonmonotonic age associations.

Annotation was conducted at the note level to capture overall narrative framing, which may obscure within-note variation. Although interrater reliability was high, stigma annotation remains inherently subjective. Annotations were performed by study investigators, raising the possibility of confirmation bias, although this risk was mitigated through detailed guidelines, coder calibration rounds, and formal reliability assessment. Independent, blinded replication and finer-grained sentence- or span-level annotation would further strengthen future corpora.

Several potentially relevant covariates could not be modeled. Provider-level factors, such as clinician specialty, training, or experience, were unavailable in MIMIC-IV. Patient-level clinical factors, including psychiatric comorbidities, substance use history, or overall illness severity, were also not incorporated and may partially confound observed associations. Notes were treated as independent observations to preserve statistical power, and clustering at the patient level was not modeled, which may result in underestimated standard errors.

NLP model development used a note-level train/test split rather than a patient-level split. Although identical notes did not appear in both the training and test sets, multiple notes from the same patient may have appeared across partitions. MSTL pretraining exclusion was also performed at the note level. Therefore, while analytic notes were excluded from the pretraining corpus, other notes from the same patients may have remained in the pretraining data. This design may introduce patient-level contextual overlap through diagnoses, demographic framing, or provider writing style. Accordingly, NLP performance and cross-domain transfer results should be interpreted as note-level held-out performance rather than patient-independent generalization.

Model evaluation was further constrained by the size of the held-out test set, particularly for subtype-specific analyses with few positive instances. Because a fixed train/test split was used to avoid direct note-level information leakage during model development, cross-validation was not applied, and performance estimates may therefore be less stable than those obtained from larger datasets. In addition, models trained on historical documentation may learn outdated linguistic patterns and may require reevaluation or recalibration before deployment in contemporary clinical environments where documentation standards and terminology have changed. In particular, subgroup fairness metrics such as TPR differences may be unstable when calculated on small denominators, and small numerical differences should be interpreted as approximate rather than precise estimates.

Fairness interventions based on equalized odds may introduce disparities in other performance metrics when baseline prevalence differs between groups. In this study, group-specific precision varied across groups, reflecting the inherent trade-offs between fairness criteria. These trade-offs are particularly relevant in auditing applications, where differences in the reliability of positive predictions may affect how model outputs are interpreted in practice.

Because misgendering represents a highly salient and often standalone form of SL, it frequently constituted the sole descriptor-level annotation within a note. Because misgendering was annotated only when the note contained self-contained evidence of affirmed gender identity, this conservative rule may have produced false negatives in notes where affirmed gender identity was ignored, and incorrect pronouns or prior names were used consistently throughout. Therefore, the observed prevalence of misgendering should be interpreted as a conservative estimate. As a result, excluding misgendering leads to a substantial reduction in descriptor-labeled notes. Although annotators were instructed to identify multiple co-occurring stigma subtypes, this distribution suggests that descriptor-level stigma may be dominated by misgendering in clinical documentation, which may influence subtype-specific comparisons.

Despite these limitations, this study establishes a reproducible framework for annotating, modeling, and evaluating SL toward GEPs in clinical text. By developing a systematically annotated dataset and demonstrating the feasibility of automated detection and fairness assessment, this work provides a foundation for larger, diverse follow-up studies aimed at improving fairness and reducing linguistic stigma in health care documentation. In addition, fairness-optimized decision thresholds were derived from the training data used for model fitting. Although this approach avoids test-set leakage, it may result in thresholds that are overfit to the training distribution and may not generalize to other datasets or clinical settings.

### Conclusion

This study shows that SL in EHR documentation is detectable and unevenly distributed, with GEPs disproportionately affected. Through manual annotation, multivariable analysis, and MSTL, we demonstrate that advanced transformer-based models exhibit reduced sensitivity to SL in GEP-related documentation. Although transfer learning improved overall performance, fairness-aware evaluation revealed persistent subgroup disparities, and these disparities remained present even when misgendering was excluded from the outcome definition. Post hoc thresholding provided a practical, model-agnostic approach for reducing subgroup disparities without retraining large models. These findings highlight the need for more inclusive datasets, refined annotation practices, and integrated fairness mechanisms to support equitable clinical NLP systems.

## Data Availability

MIMIC-IV data analyzed in this study are available through PhysioNet to credentialed users who complete the required training and sign the data use agreement [27,28]. The annotation dataset does not contain clinical note text but includes identifiers that permit linkage to MIMIC-IV records and is therefore not publicly available. The annotation dataset is available from the corresponding author on reasonable request to qualified researchers with approved MIMIC-IV access, subject to verification of PhysioNet credentials and applicable data use requirements. The code used for model training and analysis is available in the GitHub repository [47].

## Appendix

Multimedia Appendix 1 Keyword list, annotation codebook, additional regression results, and full model evaluation tables for stigmatizing language detection.

Multimedia Appendix 2 Complete held-out test set performance results for stigma subtype classification.

## Abbreviations

AIC: Akaike information criterion
BERT: Bidirectional Encoder Representations from Transformers
EHR: electronic health record
FPR: false-positive rate
GEP: gender-expansive patient
MIMIC-IV: Medical Information Mart for Intensive Care IV
MSTL: multistage transfer learning
NGEP: nongender-expansive patient
NLP: natural language processing
ONC: Office of the National Coordinator for Health Information Technology
OR: odds ratio
pp: percentage points
PR-AUC: area under the precision-recall curve
ROC-AUC: area under the receiver operating characteristic curve
SL: stigmatizing language
SST: semantic, syntactic, and task-based fine-tuning
TPR: true-positive rate
WPATH: World Professional Association for Transgender Health
