# Supplementary material for: Stigmatizing Language in Gender-Expansive Patient Records: Corpus Development, Disparity Analysis, and Natural Language Processing–Based Detection Study
Source: J Med Internet Res. 2026 Aug 27;28:e91089. doi: 10.2196/91089 (PMC13563032; doi:10.2196/91089)
Supplement: Supplementary file 1 [file jmir_v28i1e91089_app1.docx]

## Supplementary Information

## S1. Keywords Used to Filter Gender Expansive Patients (GEPs) from EHR Notes

keywords_set = ["transgender", "trans woman", "transgender woman", "trans man", "transgender man", "transwoman", "transman", "transmasculine", "transfeminine", "transperson", "transgenderperson", "gender identity", "queer", "intersex", "gender queer", "trans person", "transgender person", "transmasculine", "transfeminine", "bi gender", "twospirit", "nonbinary", "genderqueer", "genderfluid", "agender", "bigender", "pangender", "non binary", "gender fluid", "feminized", "feminize", "masculinize", "masculine", "masculinized", "gender expansive", "gender diverse", "gender variant", "gender nonconforming", "two spirit", "androgynous", "FTM", "MTF", "transexual", "transexual male", "transexual female", "queer", "enby", "assigned male", "assigned female", "assigned sex", "ASAB", "AFAB", "AMAB", "assigned gender", "AGAB", "gender identity", "deadname", "preferred gender", "preferred sex", "feminization", "gender reassignment", "gender reassigned"]

## S2. Codebook used to Annotate GEP Dataset

Task:

Analyze the following clinical note for stigmatizing language using the four predefined categories. Annotate only for stigmatizing language, words or phrases reflecting judgment, bias, disbelief, or blame from the healthcare provider toward the patient. Do not label text describing medical symptoms, diagnoses, behaviors, or phenotypes (e.g., psychosis, obesity, substance use) unless the language itself conveys a negative or moralizing tone beyond clinical description.

The definition of each category is described as follows and it serves as the basic knowledge:

1.	Descriptor – Patient’s demeanor or behavior is cast in a negative light; insinuates the patients is not being forthright or transparent; patient may be falsifying symptoms to get something they want. Examples: drug-seeking behavior; concern for secondary gain; unwilling to meet with case manager; unfortunately a poor historian; Misgendering.

2.	Descriptor - Misgendering - any incorrect pronoun, incongruent gender reference, or anatomical description inconsistent with the patient's affirmed gender. Medically necessary anatomical or organ-specific documentation was not coded as misgendering unless it was paired with an incongruent gendered label or identity-invalidating phrasing.

3.	Credibility & Obstinacy – Insinuates doubt about a patient’s stated testimony. Describes patient (or patient’s family) perspective as inflexible/difficult/entrenched, typically with respect to their intentions. Examples: adamant he doesn’t smoke; claims to see a therapist; insists on being admitted; adamantly opposed to limiting fruit intake.

4.	Compliance – paternalistic or moralizing Patient not, unlikely to, or questionably following medical advice. Examples: noncompliant; AMA; against medical advice; nonadherence; adherence to therapeutic medication is unclear; struggles with medication and follow-up compliance.

5.	Gender – either gender expansive or not gender expansive

Note: A single phrase or sentence may belong to multiple stigmatizing categories, as these categories are not mutually exclusive.

Some additional rules to help complete the task:

•	Tone and implication matter. Neutral descriptors are factual, behaviorally grounded, or describe unexpected positives without judgment. Example of Neutral: “Patient was alert and engaged.” / “Not combative or belligerent.” Negative descriptors imply dysfunction, blame, manipulation, or lack of cooperation. Example of Negative: “Frequently manipulative.” / “Refuses to answer questions.”

•	Look for implicit judgment words — e.g., “adamantly,” “claims,” “insists” — which often turn neutral phrasing into stigmatizing language.

•	If phrasing suggests the patient is a barrier to care or unreliable, label as stigmatizing (Negative).

•	If it simply describes observable behavior, label as Neutral (not stigmatizing).

•	Descriptions of behavior, appearance, or functioning will only be treated as stigmatizing when they are used to imply blame, deficiency, or moral judgment, rather than as neutral clinical observation.

•	Do not treat outdated diagnostic terms (e.g., “alcoholic cirrhosis”) as stigmatizing unless their use or context conveys judgment or blame.

•	Do not classify notes as stigmatizing if they are too subtle.

•	Use non-stigmatizing if the language refers to someone other than the patient.

•	If the note blends a family quote with provider assessment (e.g., “Mother believes patient is malingering, and that seems consistent with today’s presentation”), label based on whether the provider endorses the view negatively.

•	If the provider quotes a stigmatizing family remark purely for documentation (“He’s just crazy”), label non-stigmatizing.

•	When behavior descriptions are ambiguous (e.g., “Patient questioned the plan repeatedly”), check context: If framed as active engagement, it’s neutral. If framed as resistance, it’s stigmatizing. If unclear, label stigmatizing and explain.

•	In multi-author or templated notes, annotate only provider-authored narrative (ignore standardized EHR sections unless language reflects provider’s judgment).

•	Only provider-authored language counts as stigmatizing; statements from family members or quoted speech from others should not be labeled

•	If the provider echoes family members’ negative statements in the assessment or plan, mark those as stigmatizing under the appropriate category.

•	Language will only be labeled as stigmatizing when it directly reflects judgment, blame, disbelief, or pejorative tone within the note itself — not because it implies authority, paternalism, or hierarchy.

•	When both are present — for instance, when the note questions both the patient’s truthfulness and cooperation — use following rule: If the provider’s disbelief dominates, classify as Credibility & Obstinacy. If the provider’s behavioral judgment dominates, classify as Compliance.

•	Use “Not Stigmatizing” for neutral, clinical, or stage-based terms such as “precontemplative in recovery” or “substance use disorder.”

•	Label “Stigmatizing” when terms like “substance abuse” or “substance dependence” imply blame, moral judgment, or lack of self-control.

•	Terms such as “ensure adherence,” “encourage compliance,” or “reinforce diet restrictions” are not stigmatizing when used as clinical care plans or discharge instructions. They become stigmatizing only if framed as evidence of patient fault or disobedience, rather than logistical or clinical need.

•	When describing a patient’s lack of willingness or participation, assess whether the language conveys judgment or resistance. If phrasing merely states the patient’s choice or willingness (e.g., “patient declined” or “patient was not interested”), it is neutral. However, if wording implies defiance, resistance, or fault (e.g., “refused,” “would not cooperate,” “noncompliant”), it should be labeled as stigmatizing under the appropriate category.

•	When quoting a patient, do not label the content as stigmatizing unless the provider uses the quote in a judgmental or discrediting way. Patient quotations are considered neutral documentation when presented factually or for clinical context. However, if the provider includes or frames the quote to imply blame, ridicule, or reinforce negative stereotypes (e.g., to highlight irrationality or resistance), it should be labeled stigmatizing under the appropriate category.

## S3. Multivariable Logistic Regression Results

Models were fitted using maximum likelihood estimation, with automatic fallback to L1-regularized estimation in cases of quasi-separation.^[1]^ ORs and 95% confidence intervals (CIs) were captured, and model diagnostics included Akaike Information Criterion (AIC), McFadden’s pseudo-R², log-likelihood, and variance inflation factors (VIFs). Other (collapsed) denotes a regression category that includes Asian observations combined with other low-frequency race groups for stable estimation.

Each interaction model included all main effects and a single GEP × demographic term, with statistical significance assessed via Wald tests. Model fit was compared using AIC and pseudo-R², where an AIC reduction of ≥2.4 indicated improved fit and ≥10 indicated substantial improvement.^[2]^

| **Table S3.1.** Unadjusted model predicting any stigmatizing language (label ~ GEP) | | | |
| --- | --- | --- | --- |
| **Variable** | **Odds Ratio (OR)** | **95% CI** | **p-value** |
| Constant | 0.34 | 0.27 – 0.43 | <0.001 |
| GEP (1 = Yes) | 4.82 | 3.53 – 6.58 | <0.001 |
| Model summary note: Converged = True. | | | |

| **Table S3.2a.** Fully Adjusted Model (label ~ GEP + Demographics) | | | |
| --- | --- | --- | --- |
| **Variable** | **Odds Ratio (OR)** | **95% CI** | **p-value** |
| Constant | 0.34 | 0.24 – 0.48 | < 0.001 |
| GEP (1 = Yes) | 4.87 | 3.54 – 6.70 | < 0.001 |
| Non-English language | 1.39 | 0.65 – 2.96 | 0.391 |
| Black/African American race | 1.82 | 1.11 – 2.98 | 0.017 |
| Hispanic/Latino race | 1.63 | 0.76 – 3.51 | 0.208 |
| Other race (collapsed) | 0.66 | 0.39 – 1.12 | 0.122 |
| Age group (quartile) | 0.96 | 0.83 – 1.11 | 0.572 |
| Model summary note: AIC = 927.61, Pseudo-R² = 0.1147, Log-Likelihood = -456.81, Converged = True. | | | |

| **Table S3.2b.** Variance Inflation Factors (VIFs): | |
| --- | --- |
| **Variable** | **VIF** |
| Constant | 4.45 |
| GEP | 1.03 |
| language_grouped_NON-ENGLISH | 1.16 |
| race_grouped_BLACK/AFRICAN AMERICAN | 1.04 |
| race_grouped_HISPANIC/LATINO | 1.08 |
| race_grouped_OTHER (collapsed) | 1.17 |
| age_group | 1.04 |

| **Table S3.3.** Interaction Models (GEP × Demographic Moderation) | | | | | |
| --- | --- | --- | --- | --- | --- |
| **Model** | **Interaction** | **Odds Ratio** | **95% CI** | **p-value** | **Interpretation** |
| Model 1: GEP × Language | GEP × Non-English | 0.19 | 0.04 – 1.01 | 0.051 | No moderation by language |
| Model 2: GEP × Age | GEP × Age group | 1.08 | 0.81 – 1.43 | 0.616 | No moderation by age |
| Model 3: GEP × Race | GEP × Other (collapsed) | 0.36 | 0.14 – 0.95 | 0.038 | Reduced GEP effect in Other patients |
| Model 3: GEP × Race | GEP × Black/African American | 0.35 | 0.14 – 0.90 | 0.029 | Reduced GEP effect in Black patients |

| **Table S3.4.** Model Fit Comparison | | | | |
| --- | --- | --- | --- | --- |
| **Model** | **AIC** | **Pseudo-R²** | **Log-Likelihood** | **ΔAIC vs Baseline** |
| Baseline (S3.2a) | 927.614 | 0.115 | -456.807 | 0.000 |
| Language Interaction | 925.698 | 0.118 | -454.849 | -1.916 |
| Age Interaction | 929.362 | 0.115 | -456.681 | 1.748 |
| Race Interaction | 923.041 | 0.125 | -451.520 | -4.573 |
| Best-fitting model: Race Interaction (ΔAIC = -4.57), indicating moderate improvement with interaction terms. | | | | |

| **Table S3.5.** Sensitivity Analysis: Excluding Other (collapsed), and Unknown race | | | |
| --- | --- | --- | --- |
| **Variable** | **Odds Ratio (OR)** | **95% CI** | **p-value** |
| Constant | 0.30 | 0.21 – 0.43 | < 0.001 |
| **GEP (1 = Yes)** | **5.60** | 3.97 – 7.91 | < 0.001 |
| Non-English language | 2.38 | 0.84 – 6.79 | 0.105 |
| **Black/African American** | **1.85** | 1.12 – 3.06 | 0.016 |
| Hispanic/Latino | 1.49 | 0.68 – 3.25 | 0.315 |
| Age group (quartile) | 0.99 | 0.85 – 1.15 | 0.877 |
| Model summary note: Δ vs Baseline GEP OR = 15.0%, indicating robustness of the association; Converged = True. | | | |

| **Table S3.6.** Subtype Model 1: Credibility & Obstinacy | | | |
| --- | --- | --- | --- |
| **Variable** | **Odds Ratio (OR)** | **95% CI** | **p-value** |
| **GEP (1 = Yes)** | 2.12 | 1.43 – 3.15 | <0.001 |
| Non-English language | 0.77 | 0.28 – 2.14 | 0.619 |
| **Black/African American** | 2.14 | 1.26 – 3.64 | 0.005 |
| Hispanic/Latino | 1.37 | 0.59 – 3.22 | 0.467 |
| Other (collapsed) | 1.02 | 0.54 – 1.93 | 0.943 |
| Age group (quartile) | 0.88 | 0.74 – 1.05 | 0.151 |
|  | | | |

| **Table S3.7.** Subtype Model 2: Compliance | | | |
| --- | --- | --- | --- |
| **Variable** | **Odds Ratio (OR)** | **95% CI** | **p-value** |
| **GEP (1 = Yes)** | 2.81 | 1.89 – 4.16 | < 0.001 |
| Non-English language | 1.54 | 0.63 – 3.76 | 0.348 |
| **Black/African American** | 2.61 | 1.57 – 4.34 | < 0.001 |
| Hispanic/Latino | 1.83 | 0.85 – 3.96 | 0.123 |
| Other (collapsed) | 0.60 | 0.30 – 1.20 | 0.151 |
| Age group (quartile) | 0.84 | 0.70 – 0.99 | 0.041 |
|  | | | |

| **Table S3.8.** Subtype Model 3: Descriptors | | | |
| --- | --- | --- | --- |
| **Variable** | **Odds Ratio (OR)** | **95% CI** | **p-value** |
| **GEP (1 = Yes)** | 7.37 | 5.16 – 10.53 | <0.001 |
| Non-English language | 0.99 | 0.40 – 2.47 | 0.991 |
| **Black/African American** | 0.95 | 0.56 – 1.61 | 0.846 |
| Hispanic/Latino | 1.03 | 0.47 – 2.24 | 0.939 |
| Other (collapsed) | 0.58 | 0.32 – 1.03 | 0.063 |
| Age group (quartile) | 0.92 | 0.79 – 1.07 | 0.285 |
|  | | | |

| **Table S3.9.** Subtype Model 4: Descriptors excluding misgendering | | | |
| --- | --- | --- | --- |
| **Variable** | **Odds Ratio (OR)** | **95% CI** | **p-value** |
| **GEP (1 = Yes)** | 2.33 | 1.61 – 3.38 | <0.001 |
| Non-English language | 1.02 | 0.39 – 2.63 | 0.975 |
| **Black/African American** | 1.44 | 0.85 – 2.43 | 0.176 |
| Hispanic/Latino | 0.65 | 0.26 – 1.65 | 0.365 |
| Other (collapsed) | 0.71 | 0.38 – 1.32 | 0.277 |
| Age group (quartile) | 0.90 | 0.76 – 1.06 | 0.211 |
|  | | | |

| **Table S3.10.** Unadjusted model predicting any stigmatizing language excluding misgendering (label ~ GEP) | | | |
| --- | --- | --- | --- |
| **Variable** | **Odds Ratio (OR)** | **95% CI** | **p-value** |
| Constant | 0.34 | 0.27 – 0.43 | <0.001 |
| GEP (1 = Yes) | 2.10 | 1.54 – 2.85 | <0.001 |
| Model summary note: Converged = True. | | | |

| **Table S3.11a.** Fully Adjusted Model excluding misgendering (label ~ GEP + Demographics) | | | |
| --- | --- | --- | --- |
| **Variable** | **Odds Ratio (OR)** | **95% CI** | **p-value** |
| Constant | 0.33 | 0.23 – 0.46 | < 0.001 |
| GEP (1 = Yes) | 2.12 | 1.54 – 2.91 | < 0.001 |
| Non-English language | 1.39 | 0.66 – 2.91 | 0.384 |
| Black/African American race | 2.33 | 1.46 – 3.72 | < 0.001 |
| Hispanic/Latino race | 1.77 | 0.86 – 3.63 | 0.118 |
| Other race (collapsed) | 0.80 | 0.47 – 1.34 | 0.390 |
| Age group (quartile) | 0.94 | 0.82 – 1.09 | 0.435 |
| Model summary note: AIC = 934.05, Pseudo-R² = 0.0424, Log-Likelihood = -460.03, Converged = True. | | | |

| **Table S3.11b.** Variance Inflation Factors (VIFs) excluding misgendering: | |
| --- | --- |
| **Variable** | **VIF** |
| Constant | 4.451 |
| GEP | 1.030 |
| language_grouped_NON-ENGLISH | 1.159 |
| race_grouped_BLACK/AFRICAN AMERICAN | 1.036 |
| race_grouped_HISPANIC/LATINO | 1.076 |
| race_grouped_OTHER (collapsed) | 1.173 |
| age_group | 1.037 |

| **Table S3.12.** Interaction Models excluding misgendering (GEP × Demographic Moderation) | | | | | |
| --- | --- | --- | --- | --- | --- |
| **Model** | **Interaction** | **Odds Ratio** | **95% CI** | **p-value** | **Interpretation** |
| Model 1: GEP × Language | GEP × Non-English | 0.24 | 0.04 – 1.47 | 0.124 | No moderation by language |
| Model 2: GEP × Age | GEP × Age group | 1.02 | 0.77 – 1.35 | 0.910 | No moderation by age |
| Model 3: GEP × Race | GEP × Black/African American | 0.61 | 0.24 – 1.56 | 0.304 | No moderation by race |

| **Table S3.13.** Model Fit Comparison excluding misgendering | | | | |
| --- | --- | --- | --- | --- |
| **Model** | **AIC** | **Pseudo-R²** | **Log-Likelihood** | **ΔAIC vs Baseline** |
| Baseline (S3.11a) | 934.054 | 0.042 | -460.027 | 0.00 |
| Language Interaction | 933.416 | 0.045 | -458.708 | -0.638 |
| Age Interaction | 936.041 | 0.042 | -460.021 | 1.987 |
| Race Interaction | 934.746 | 0.048 | -457.373 | 0.692 |
| Best-fitting model: Language Interaction (ΔAIC = -0.64), indicating models equivalent. | | | | |

| **Table S3.14.** Sensitivity Analysis excluding misgendering: Excluding Other (collapsed), and Unknown race | | | |
| --- | --- | --- | --- |
| **Variable** | **Odds Ratio (OR)** | **95% CI** | **p-value** |
| Constant | 0.29 | 0.20 – 0.41 | < 0.001 |
| **GEP (1 = Yes)** | **2.33** | 1.66 – 3.28 | < 0.001 |
| Non-English language | 2.08 | 0.76 – 5.71 | 0.154 |
| **Black/African American** | **2.36** | 1.47 – 3.78 | <0.001 |
| Hispanic/Latino | 1.68 | 0.81 – 3.49 | 0.164 |
| Age group (quartile) | 0.98 | 0.85 – 1.15 | 0.842 |
| Model summary note: Δ vs Baseline GEP OR = 9.9%, showing stable association; Converged = True. | | | |

## S4. Complete Evaluation of Model Performance Across Stigma Subtypes

### S4.1 Summary of Model Performance on Stigma Subtypes

To evaluate subtype-specific classification, all models were trained independently for each stigma subtype (Credibility & Obstinacy, Compliance, Descriptors, and Misgendering), encompassing both traditional machine-learning classifiers (Logistic Regression, SVM, Random Forest, Naïve Bayes) and transformer-based architectures (BERT, ClinicalBERT, Longformer, and MSTL-Longformer). Training used an 80/20 split of the GEP-inclusive dataset, yielding 151 test notes per subtype. Each model was evaluated using accuracy, precision, recall, F1, and AUC to capture both discriminative performance and sensitivity to minority (stigmatizing) instances.

Across the held-out test set, substantial class imbalance was observed for all subtypes. For Credibility & Obstinacy, 25 notes (16.6%) contained stigmatizing language, whereas 126 (83.4%) were non-stigmatizing. The same distribution was observed for Compliance, with 25 (16.6%) stigmatizing and 126 (83.4%) neutral instances. Descriptors exhibited a higher prevalence of stigma, with 51 (33.8%) stigmatizing and 100 (66.2%) non-stigmatizing notes. Misgendering remained moderately imbalanced, with 32 (21.2%) containing misgendering expressions and 119 (78.8%) correctly gendered. These distributions demonstrate that the majority of notes across subtypes were non-stigmatizing, underscoring the rarity of overtly biased language in clinical documentation and the need for evaluation metrics beyond accuracy alone.

As summarized in Table S4.1, model performance varied across subtypes and optimization objectives. Among pretrained transformer models, the MSTL-Longformer consistently produced the strongest results for Credibility & Obstinacy and Descriptors, demonstrating that multi-stage transfer learning substantially improves generalization for these linguistically complex and context-dependent forms of bias. When considering models selected by accuracy, the standard Longformer (accuracy optimized) checkpoint achieved the best performance for Compliance, while ClinicalBERT (accuracy optimized) performed best for Misgendering. Under F1-selected criteria, which prioritize balanced sensitivity and precision under class imbalance, the overall pattern remained similar: MSTL-Longformer again yielded the top performance for Credibility & Obstinacy and Descriptors, and Longformer (F1 optimized) improved recall for Misgendering relative to its accuracy optimized version. Across all subtypes, AUC values exceeded 0.73, reflecting stable discriminatory capacity even under pronounced class imbalance.

| **Table S4.1.** Best-Performing Models by Stigma Subtypes | | | | | | |
| --- | --- | --- | --- | --- | --- | --- |
| Subtype | Model | Accuracy | Precision | Recall | F1 | AUC |
| Best Models by Accuracy | |  |  |  |  |  |
| Credibility & Obstinacy | Longformer (MSTL) | 0.9139 | 0.7308 | 0.7600 | 0.7451 | 0.9311 |
| Compliance | Longformer (Accuracy) | 0.9205 | 0.7407 | 0.8000 | 0.7692 | 0.9419 |
| Descriptors | Longformer (MSTL) | 0.8212 | 0.7143 | 0.7843 | 0.7477 | 0.8380 |
| Misgendering | ClinicalBERT (Accuracy) | 0.8411 | 0.7500 | 0.3750 | 0.5000 | 0.7634 |
| Best Models by F1 | |  |  |  |  |  |
| Credibility & Obstinacy | Longformer (MSTL) | 0.9139 | 0.7308 | 0.7600 | 0.7451 | 0.9311 |
| Compliance | Longformer (Accuracy) | 0.9205 | 0.7407 | 0.8000 | 0.7692 | 0.9419 |
| Descriptors | Longformer (MSTL) | 0.8212 | 0.7143 | 0.7843 | 0.7477 | 0.8380 |
| Misgendering | Longformer (F1) | 0.7682 | 0.4681 | 0.6875 | 0.5570 | 0.7532 |

*Note.* “Accuracy” and “F1” in parentheses indicate each model’s optimization objective. MSTL denotes the multi-stage transfer learning framework. Longformer (MSTL) is included for benchmarking.

Collectively, these results show that multi-stage transfer learning (MSTL) demonstrates the greatest performance for linguistically subtle subtypes such as Credibility & Obstinacy and Descriptors, whereas simpler accuracy optimized architectures suffice for more lexically explicit categories like Compliance and Misgendering. The observed divergence between accuracy and F1 optimized outcomes underscores the influence of severe class imbalance on model generalization and highlights the importance of evaluating models using complementary performance metrics when developing NLP systems for stigmatizing language detection in EHRs. The full performance of all models tested in this section can be found in supplementary information S4.2.

### S4.2 Complete Evaluation Metrics for Stigma Subtype Classification Across Models

This section presents the comprehensive performance results for all models evaluated on subtype-specific stigma classification tasks. Traditional machine-learning classifiers (Logistic Regression, Support Vector Machine, Random Forest, Naïve Bayes) and transformer-based architectures (BERT, ClinicalBERT, Longformer, and Multi-Stage Transfer Learning (MSTL) Longformer) were assessed under both accuracy-optimized and F1-optimized configurations. Each model was independently trained for one of the four stigma subtypes, including Credibility & Obstinacy, Compliance, Descriptors, and Misgendering. They are evaluated using an 80/20 split of the GEP-inclusive corpus.

Performance metrics include Accuracy, Precision, Recall, F1, and AUC, reported separately for the overall dataset and for subgroup partitions (GEP = 0, GEP = 1) to evaluate fairness and generalization. The results highlight how optimization objectives (accuracy vs. F1) and model complexity (traditional vs. transformer) interact with data imbalance across subtypes. The complete model evaluation results are provided in Multimedia Appendix 2.

Table Legend:

- Optimization: Indicates whether the model was optimized for overall accuracy or F1 score during validation.
- Family: Model family indicates Traditional (LR, NB, RF, SVM) or Transformer (BERT, ClinicalBERT, Longformer, MSTL-Longformer).
- Subtype: One of four stigma subtypes.
- Group: Evaluation subset (Overall, GEP = 0, GEP = 1).
- N: Number of test instances within each group.
- Accuracy, Precision, Recall, F1, AUC: Standard classification metrics calculated from the test set.

Abbreviations:

- LR: Logistic Regression
- NB: Naïve Bayes
- RF: Random Forest
- SVM: Support Vector Machine
- MSTL: Multi-Stage Transfer Learning

### S5. Note-Level Overlap Between Non-Misgendering Descriptors and Misgendering

**Figure S5.** Note-level overlap between non-misgendering descriptors and misgendering among GEP notes. The schematic shows that 102 GEP notes contained non-misgendering stigmatizing descriptors, including 51 notes with non-misgendering descriptors only and 51 notes with both non-misgendering descriptors and misgendering. An additional 101 notes contained misgendering without a non-misgendering descriptor, yielding 203 descriptor-labeled GEP notes when misgendering was included.

## S6. Complete Evaluation of Model Performance Across Stigma Subtypes

This section presents detailed group-specific performance metrics for all evaluated models under both outcome definitions (including and excluding misgendering), as summarized in Table S6. Metrics are reported separately for gender-expansive (GEP) and non-gender-expansive (NGEP) patients to complement the fairness analysis in the main text.

In addition to true positive rate (TPR) and false positive rate (FPR), we report precision (positive predictive value) to illustrate trade-offs that arise when baseline prevalence differs between groups. These results provide a more complete characterization of model performance and provide context for the fairness analysis discussed in the main manuscript. All rows report the baseline configuration (global threshold = 0.5). No row reports the accuracy-optimized (best-accuracy) or fairness-optimized (best-fair) configuration.

| **Table S6.** Group-Specific Performance Metrics by Model and Outcome Definition | | | | | | | |
| --- | --- | --- | --- | --- | --- | --- | --- |
| Model | Outcome | Precision (NGEP) | Precision (GEP) | TPR (NGEP) | TPR (GEP) | FPR (NGEP) | FPR (GEP) |
| Longformer: GEP | Including misgendering | 0.5385 | 0.7200 | 0.7000 | 0.8000 | 0.2034 | 0.5185 |
| Longformer: SST+GEP | Including misgendering | 0.7391 | 0.8333 | 0.8500 | 0.7778 | 0.1017 | 0.2593 |
| BERT: GEP | Including misgendering | 0.7222 | 0.7255 | 0.6500 | 0.8222 | 0.0847 | 0.5185 |
| ClinicalBERT: GEP | Including misgendering | 0.5357 | 0.7727 | 0.7500 | 0.7556 | 0.2203 | 0.3704 |
| Longformer: GEP | Excluding misgendering | 0.6296 | 0.7188 | 0.8500 | 0.7419 | 0.1695 | 0.2195 |
| Longformer: SST+GEP | Excluding misgendering | 0.8000 | 0.8621 | 0.8000 | 0.8065 | 0.0678 | 0.0976 |
| BERT: GEP | Excluding misgendering | 1.0000 | 0.7692 | 0.4000 | 0.6452 | 0.0000 | 0.1463 |
| ClinicalBERT: GEP | Excluding misgendering | 0.6429 | 0.7188 | 0.4500 | 0.7419 | 0.0847 | 0.2195 |
| *Note.* TPR: true positive rate; FPR: false positive rate; NGEP: non-gender-expansive patients; GEP: gender-expansive patients. | | | | | | | |

Reference

1	Dreiseitl S, Ohno-Machado L. Logistic regression and artificial neural network classification models: a methodology review. *Journal of Biomedical Informatics*. 2002;35:352–9. doi: 10.1016/S1532-0464(03)00034-0

2	Burnham KP, Anderson DR, Huyvaert KP. AIC model selection and multimodel inference in behavioral ecology: some background, observations, and comparisons. *Behav Ecol Sociobiol*. 2011;65:23–35. doi: 10.1007/s00265-010-1029-6
